# An Automated Image-Based Dietary Assessment System for Mediterranean Foods

**DOI:** 10.1109/OJEMB.2023.3266135

**Published:** 2023-04-13

**Authors:** Fotios S. Konstantakopoulos, Eleni I. Georga, Dimitrios I. Fotiadis

**Affiliations:** Unit of Medical Technology and Intelligent Information Systems, Materials Science and Engineering DepartmentUniversity of Ioannina 37796 GR 45110 Ioannina Greece; Biomedical Research InstituteFORTH, University of Ioannina 37796 GR 45110 Ioannina Greece

**Keywords:** Mediterranean diet, dietary assessment system, computer vision, deep learning, stereo-vision

## Abstract

*Goal*: The modern way of living has significantly influenced the daily diet. The ever-increasing number of people with obesity, diabetes and cardiovascular diseases stresses the need to find tools that could help in the daily intake of the necessary nutrients. *Methods:* In this paper, we present an automated image-based dietary assessment system of Mediterranean food, based on: 1) an image dataset of Mediterranean foods, 2) on a pre-trained Convolutional Neural Network (CNN) for food image classification, and 3) on stereo vision techniques for the volume and nutrition estimation of the food. We use a pre-trained CNN in the Food-101 dataset to train a deep learning classification model employing our dataset Mediterranean Greek Food (MedGRFood). Based on the EfficientNet family of CNNs, we use the EfficientNetB2 both for the pre-trained model and its weights evaluation, as well as for classifying food images in the MedGRFood dataset. Next, we estimate the volume of the food, through 3D food reconstruction of two images taken by a smartphone camera. The proposed volume estimation subsystem uses stereo vision techniques and algorithms, and needs the input of two food images to reconstruct the point cloud of the food and to compute its quantity. *Results:* The classification accuracy where true class matches with the most probable class predicted by the model (Top-1 accuracy) is 83.8%, while the accuracy where true class matches with any one of the 5 most probable classes predicted by the model (Top-5 accuracy) is 97.6%, for the food classification subsystem. The food volume estimation subsystem achieves an overall mean absolute percentage error 10.5% for 148 different food dishes. *Conclusions:* The proposed automated image-based dietary assessment system provides the capability of continuous recording of health data in real time.

## I. Introduction

A daily healthy diet and a daily intake of essential nutrients can significantly affect the modern lifestyle as they prevent malnutrition in all its forms, but also a wide range of nutrition-related diseases. Chronic noninfective diseases, such as obesity, diabetes and cardiovascular diseases may be affected either positively or negatively by an individual's diet. Diabetes mellitus is considered a group of metabolic disorders characterized by a high blood sugar level (glucose) and has become a common disease of the modern way of life [1]. To date about 537 million adults live with diabetes, while this number is projected to rise to 783 million by 2045. Moreover, diabetes has been associated with 6.7 million deaths globally in 2021 [2]. Cardiovascular diseases (CVDs) are a group of heart or blood vessels disorders and are the principal cause of death worldwide, approximately 17.9 million deaths each year. CVDs representing 32% of all deaths regardless the cause [3]. Obesity and overweight is a complex disease involving an excessive amount of body fat that may impair health. As 39 million children under the age of five in 2020 were overweight or obese, one could say that obesity has reached epidemic proportions worldwide [4]. Over time, obesity leads to many serious health problems such as heart diseases, stroke, type 2 diabetes and some types of cancer [5]. Over the past two years the whole world has been plagued by the pandemic called coronavirus disease (COVID-19), which causes primarily seriously respiratory problems [6]. COVID-19 is characterized, among others, mainly by pneumonia, lymphopenia, exhausted lymphocytes and cytokine storm and has led so far to millions of deaths worldwide [7]. Recent studies [8], [9] show that proper nutrition should be taken into account in patients who have been admitted to intensive care units (ICUs) due to the SARS-CoV-2 virus.

Nowadays, with Artificial Intelligence (AI), the Internet of Things (IoT) and computer vision becoming omnipresent technologies, individuals are allowed to use food applications to monitor and record their daily diet [10]. The large number of these applications and their increasing use, has made them more popular, compared to traditional methods of calculating nutritional composition. Important parts in nutrition apps are the food classification systems, the volume estimation systems, as well as the food image and the nutrition databases.

The food image dataset is the key to creating a highly accurate model for a dietary assessment system. These datasets can be characterized by the number of food classes [11] and the total number of images they include [12], the type of cuisine [13], the quality of the images, the source of the images [14] and by their use (for training or evaluation of the food classification system, or used only for the evaluation of the food volume estimation system) [15].

Image classification, which is a subset of AI and computer vision, is the process of defining and labeling groups of pixels or vectors within an image based on precise rules Food classification systems are responsible for the identification of the food class to which a food image belongs. Existing food image classification approaches can be divided into two main categories: (1) traditional machine learning approaches [16], and (2) deep learning approaches [17]. The creation of more accurate classification models along with the increased computing capabilities and the ability to train deep learning models through the graphic processing unit (GPU), imposed deep learning approaches for the classification of food images in the last years. The existence of large food datasets has also played an important role in this choice, as the effectiveness of deep learning approaches depends directly on the number of images in the dataset. The metrics used to evaluate food classification models are the top-1 and top-5 accuracy. Top-1 accuracy is the accuracy where true class matches with the most probable classes predicted by the model, while top-5 accuracy is the accuracy where true class matches with any one of the five most probable classes predicted by the model.

The final step in a food dietary assessment system comprises the estimation of foods’ quantity and the analysis of their nutritional composition, such as carbohydrates, proteins, fats and total calories. Accurate estimation of the amount of food, assumes that the previous step of food classification has been accomplished correctly. The existing approaches in food image volume estimation, can be divided into five main categories: stereo-based approaches [18], depth camera approaches [19], pre-build shape templates approaches [20], perspective transformation approaches [21] and deep learning approaches [22]. The food volume estimation is a demanding process which in most cases requires a specific number of images and a specific way of taking them, a controlled environment and in many cases dedicated cameras for capturing food images.

In this study, we present a fully automated image-based dietary assessment system. We report the characteristics of the food image dataset and the methods used for classifying the content of food images and estimating the food volume. First, we present a new food image dataset representing the Mediterranean cuisine which enables the development and evaluation of both computational modelling subsystems. This is the first study in this field concerning Mediterranean foods and, in addition to this, the number of foods used to evaluate the food volume estimation system is greater than those reported in the relevant literature [23]. Second, unlike most food-image classification studies, we leverage a model pre-trained on food imaging-data instead of the non-specific ImageNet dataset. In particular, we use a Convolutional Neural Network (CNN) pre-trained on the Food-101 dataset [11]. Third, the food volume estimation relies on the 3D-reconstruction of the food and its volume computation from two distinct images via stereo vision techniques. Fourth, the Greek Food Composition Dataset by the Hellenic Health Foundation [24] allow us to infer food macronutrients, i.e., carbohydrates, proteins and fats.

This study is a continuation of our recent work in this field [25], [26], [27] synthesizing improved versions of the employed methods in an integrated fully automated system and providing an enhanced evaluation of their predictive performance. First, we progressively built the MedGRFood dataset by increasing both the number of represented food classes and the number of images per class; its complete version is provided herein. Second, the architecture of the EfficientNetB2-based food classification subsystem, as compared with the one presented in [25], has been refined by utilising a pretrained model on a food images-specific dataset (i.e., the Food-101 dataset) which is, subsequently, fine-tuned on the complete version of the MedGRFood dataset; these modifications were translated into more accurate predictions. Though the methods of the food volume estimation subsystem are similar to those specified in [26], herein, we demonstrate their robustness by testing and verifying the reproducibility of the results using the updated dataset comprising 10000 images (vs 5000 images in its previous version). In addition, the functionality of the latter subsystem is enhanced by adding a subcomponent indexing the recognised food into the Greek Food Composition Dataset and calculating its macronutrients based on the estimated volume. In [27], we presented an overview of the entire system as built upon a previous version of the dataset; nonetheless, herein, we transparently report all technical details required for reproducing the underlying methods and results.

The primary intended use of the proposed system lies in the context of digital-based dietary assessment interventions, e.g., as integrated into a dietary assessment application aiming at improving a person's eating habits or into an electronic health record system aiming at monitoring and prescribing the diet of patients. In addition, the output of the proposed system, i.e., macronutrients, can make up the input of predictive models of health outcomes affected by one individual's diet, e.g., blood glucose prediction models.

## II. Materials and Methods

**Fig. 1.**
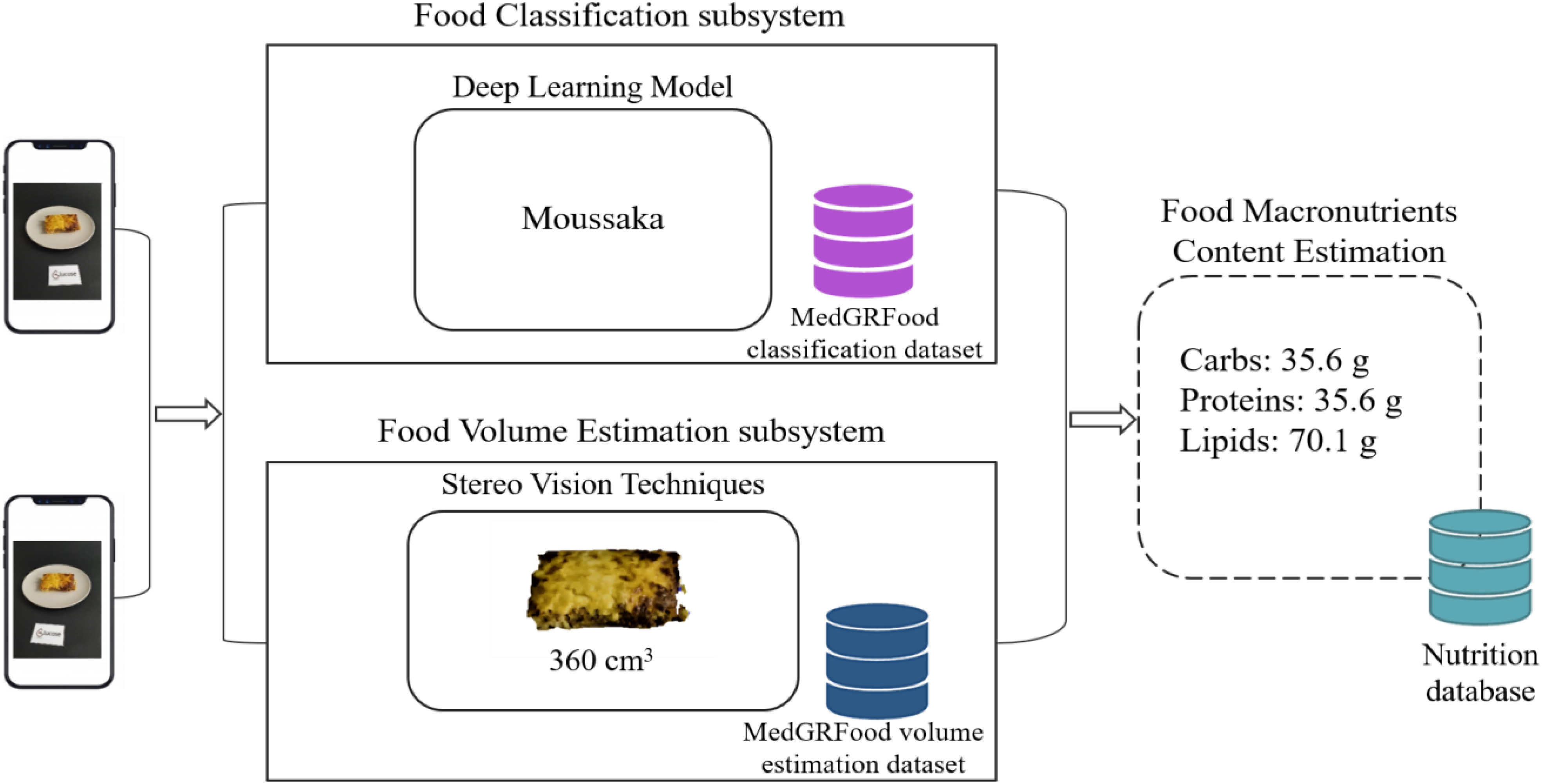
The proposed dietary assessment system.

The proposed dietary assessment system needs two food images to calculate the nutritional composition of the food. Initially, using a smartphone camera, the images are taken with a shooting angle of about 45 degrees from the vertical axis of food, and at a distance of about 40−50 cm. A reference card placed next to the dish helps to estimate the scale of the captured images. Then, the food classification subsystem, using deep learning techniques, recognizes the class of the food. At the same time, the food volume estimation subsystem, using stereo vision techniques creates a 3D reconstruction of the food and estimates its quantity. An appropriate food image database was created for the training and evaluation of the two subsystems. The image database consists of two sub-datasets: (a) an image dataset appropriate for deep learning classification models, and (b) an image dataset appropriate for food volume estimation systems based on stereo vision techniques. Finally, knowing the class of food and its volume, using the USDA database [28] and the Greek Food Composition Dataset by the Hellenic Health Foundation, we compute the nutritional composition of the capturing food. The main parts of the proposed dietary assessment system are: (i) the food image dataset and food nutrition database, (ii) the classification subsystem, and (iii) the volume estimation subsystem are shown in Fig. 1. The following subsections describe and analyze the main parts, techniques and algorithms of the proposed dietary assessment system.

### A. MedGRFood Image Dataset

The Mediterranean diet is a well-known healthy dietary pattern which is based on the traditional cuisines of Greece, Italy, Spain and other countries that border the Mediterranean Sea. Plant-based foods, such as vegetables, legumes, fruits, nuts and seeds, are the foundation of the diet. Olive oil is the main source of added fat. Moreover, fish, seafood, dairy (e.g., yogurt, cheese) and poultry are included in moderation while red meat and sweets are eaten only occasionally [29]. Adoption of the Mediterranean diet has been associated with a reduced risk of developing chronic diseases, such as diabetes, CVDs, obesity and even cancer [30], [31]. Therefore, we collected food images of the Mediterranean cuisine and we created the MedGRFood dataset, which are classified into eight food categories, based on the Greek Food Composition Dataset by the Hellenic Health Foundation. More specifically, the food categories are the following: (i) Milk, dairy products or milk substitutes, (ii) Egg or egg products, (iii) Meat or meat products, (iv) Seafood or related products, (v) Grain or grain products, (vi) Vegetable or vegetable products, (vii) Sugar or sugar products, and (viii) Miscellaneous food products. The MedGRFood dataset includes two sub-datasets: (a) the first sub-dataset consists of 51,840 food images which belong to 160 food classes, with 324 images per class and is appropriate for food classification systems, and (b) the second sub-dataset is appropriate for food volume estimation systems and consists of 10,000 food images which belong to 190 food classes. In the first sub-dataset most of the images have been collected from the web, while the rest have been taken under specific conditions, completing the required number of images per food class for a balanced dataset. This is a labelled dataset that contains good quality images, making it ideal for training and evaluating deep learning classification models. In the second sub-dataset all images have been collected under specific conditions (lighting and capturing angle conditions) with a reference card next to the plate and it contains high quality images with known weight of food. For each food class there are at least five different dishes; and for each dish at least two pairs of images are captured, suitable for stereo vision analysis and Structure from Motion (SfM) systems.

### B. Food Classification Subsystem

To create the food recognition model we used as base model an existing CNN classification model, that belongs to the EfficientNet [32] model family. First, we trained the CNN model in the Food-101 dataset and then we trained the new food classification model using the MedGRFood dataset. Moreover, transfer learning, fine tuning and data augmentation techniques were applied to improve the top-1 and top-5 accuracy and to reduce the loss of the food classification model. We have chosen the Food-101 image dataset due to the large number of food images it contained in each food class, allowing this way the pre-trained classification model to be train of and adjust better the weights of the model's layers to images containing food items.

#### 1) EfficientNet

EfficientNet is a convolutional neural network architecture and scaling method that uniformly scales all dimensions of depth, width and resolution using a compound coefficient. In contrast to the conventional practice of arbitrarily scaling these factors, the EfficientNet scaling method uniformly scales network depth, width and resolution with a set of fixed scaling coefficients. The depth corresponds to the number of layers in the CNN. The width is related to the number of neurons in a layer, or more relevantly to the number of filters in a convolutional layer. The resolution is the height and width of the input image. Increasing the depth, by adding more convolutional layers, allows the network to learn more complicated features. However, deeper networks become difficult to train and require more computational power. Scaling the width of the CNNs allows layers to learn more detailed features. Higher image resolution provides a greater detail of image details, improving the model's ability to detect and analyze relatively smaller objects and export finer patterns [32]. In this study we use the EfficientNetB2 as base model for the food image classification subsystem.

#### 2) System Architecture

**Fig. 2.**
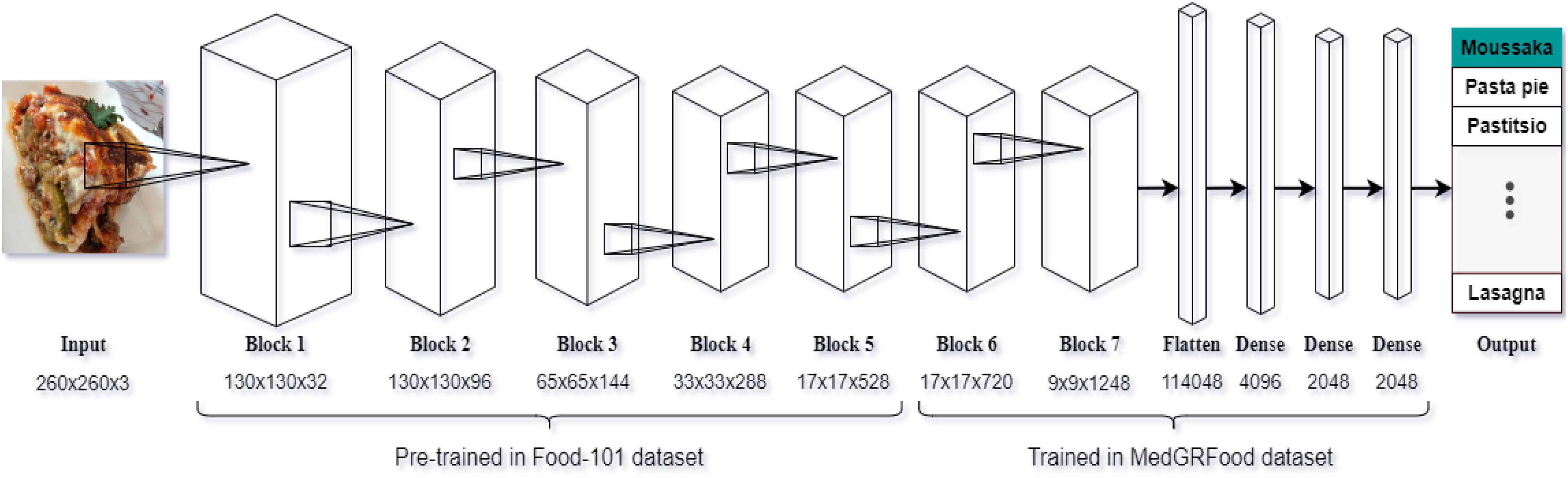
The architecture of the proposed classification model.

The architecture of food classification subsystem consists of seven CNN block layers based on EfficientNet-B2 deep learning model and three blocks of fully connected activation, batch normalization and drop-out layers. Each of the seven blocks have a varying number of sub-blocks. The total number of layers is 349 for the classification model. The architecture of the baseline network uses a mobile inverted bottleneck convolution similar to MobileNet V2 [33] but is slightly larger due to the increase in Floating-point Operations per Second (FLOPS). The size of the input image is 260x260x3 and the total number of the models’ trainable parameters are 486,359,066. The architecture of the proposed classification model is shown in Fig. 2. We use the multi-class cross-entropy loss function for the food classification problem. The cross-entropy calculates a score that summarizes the average difference between the actual and predicted probability distributions for all classes in the problem. The score is minimized and a perfect cross-entropy value is 0. The model has been trained for 250 epochs using the stochastic gradient descent (SGD) optimizer [34]. Finally, we chose a scaled learning rate with initial value 0.001 and final value 0.0000001. The learning rate decreases by a factor 0.9 when the validation loss stops improving for three epochs. Finally, for the activation function we used the Swish [35].

#### 3) Transfer Learning and Fine-Tuning

Deep learning algorithms, so far, have been designed to work individually. These algorithms are trained to solve specific tasks and must be rebuilt from scratch as soon as the distribution of feature-space changes. Transfer learning is the concept of overcoming the isolated learning paradigm and leveraging the knowledge gained for a task to solve related ones. Moreover, it is considered a popular method in machine learning because it allows the user to build accurate models in a timesaving way [36]. In deep neural networks, the initial layers are used to capture general features and detect simpler patterns, such as edges, corners and shape features, while the subsequent layers focus more on specific dataset features and more complex patterns that they detect. Taking advantage of this property, the intuition behind transfer learning is that it focuses on storing the knowledge gained in solving a problem and applying it to a different but related problem. Throughout the related literature, the training of the food classification model is done either from scratch or transfer learning is applied, using a pre-trained CNN model in the ImageNet LSVRC-2012 dataset. In this study we apply the transfer learning technique, but we use a pre-trained CNN model in the Food-101 dataset and not in ImageNet. Knowing that the early layers extract and learn general features (such as edges and simple textures) while the later layers extract and learn detailed or high-level features (such as more complex textures and patterns), we take advantage of the related images with our own dataset and their large number of Food-101 images, by transferring knowledge to our own task.

Fine-tuning involves unfreezing some layers of the CNN model for feature extraction and jointly training the newly added part of the model and these top layers. We use the fine-tuning technique, in which the first layers of the recognition model are frozen, transferring to these layers the weights of the pre-trained model mentioned above, while the last layers of model are unfreezing and re-trained on the new data with a very low learning rate. This can potentially achieve meaningful improvements by incrementally adapting the pre-trained features to the new data. Initially, in the proposed CNN model, we unfreeze the last two blocks of network layers. Then, we add three blocks of fully connected, activation and drop-out layers on the base EfficientNet-B2. Finally, the unfreezing layers and the additional layers are trained in MedGRFood dataset. These added layers further increase the performance of the proposed model and prevent overfitting problem.

#### 4) Data Augmentation

Data augmentation is based on techniques used to enhance the volume and the diversity of training data, by applying random but realistic transformations of existing data. Data augmentation acts as a regularizer, makes the model more robust to slight variations, helps to reduce overfitting and improves the performance when training a CNN model [37]. In the field of image classification, these are done to utilize the full power of the CNN, which is able to capture translational invariance. This translational invariance is what makes image classification such a difficult task. Our goal is to train a set of food images which is as representative as possible in reality, increasing the number of images that will be trained. Therefore, geometric transformation such as cropping, flipping, zooming and rotation techniques are applied to increase the amount and the variety of food images in the training data.

### C. Food Volume Estimation Subsystem

**Fig. 3.**
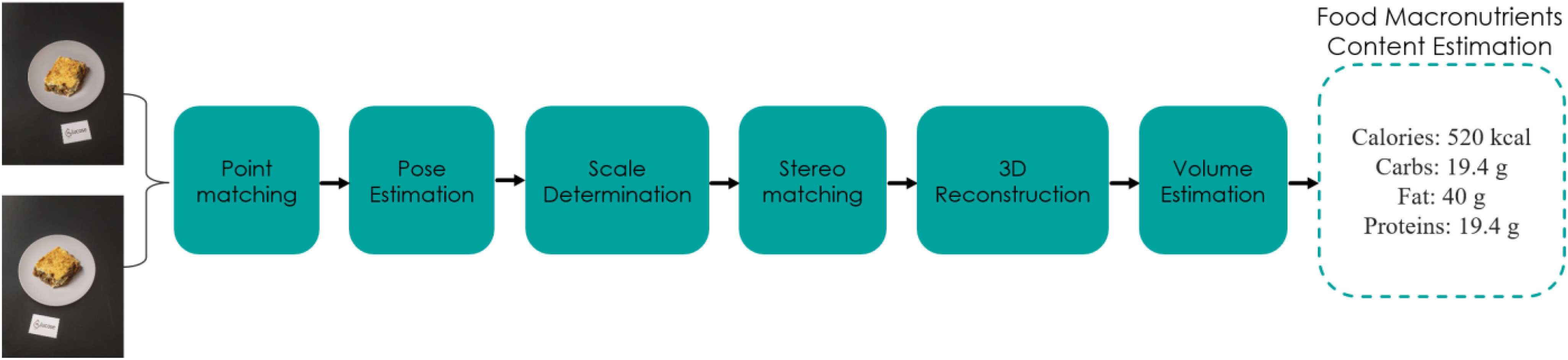
Proposed volume and nutrition estimation subsystem pipeline.

Initially, for the estimation of food volume and for its 3D reconstruction, some assumptions need to be made: i) the smartphone's camera is calibrated and we know the intrinsic camera matrix, ii) the food volume estimation subsystem needs two images of food to calculate its volume, iii) the two images are taken with a slight shifting of the camera to the right when capturing the second image, iv) the images are taken at a distance of about 40−50 cm from the dish, and with a shooting angle of about 45 degrees from the vertical axis of food, v) the type of dish used should be shallow, and vi) a reference card of known dimensions (8.5 × 5.5 cm) is placed next to the dish. Fig. 3 shows the pipeline and the major steps of the proposed methodology.

#### 1) Image Pre-Processing and Point Matching

**Fig. 4.**
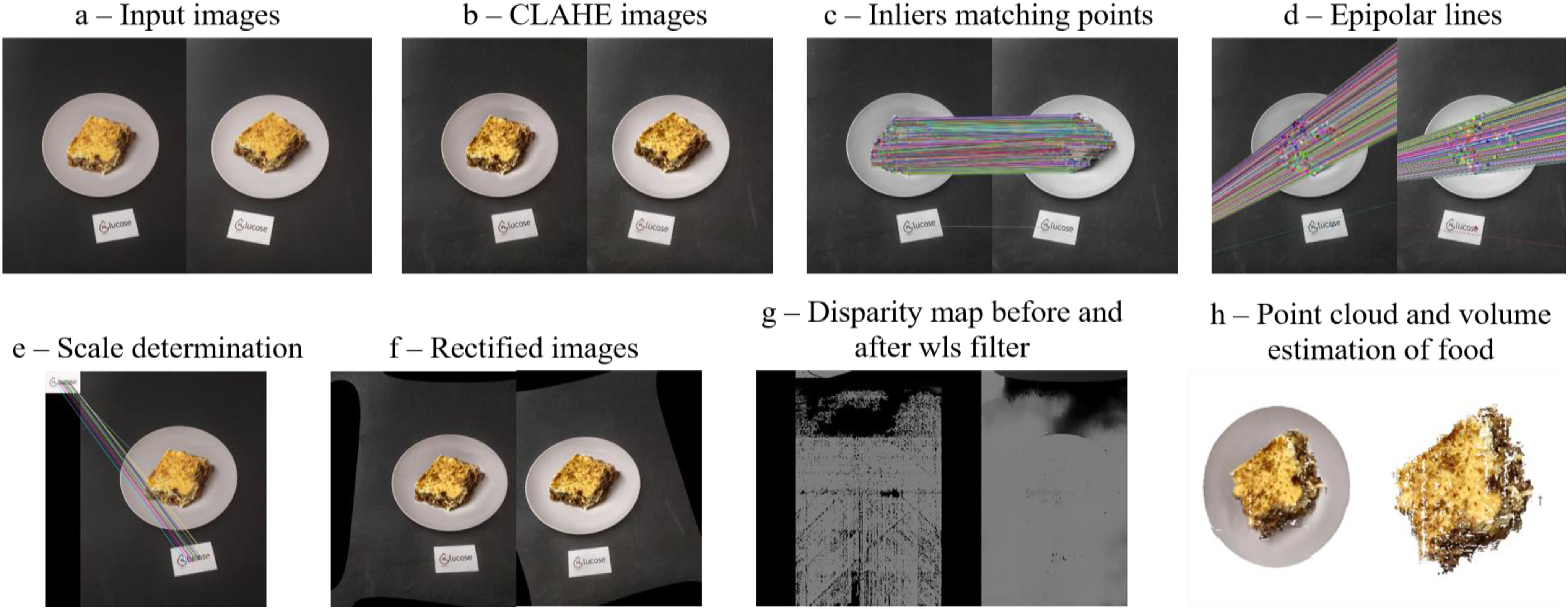
Example of the proposed methodology for the dietary assessment subsystem.

The first step of the proposed methodology is to improve the quality of the input images by applying the CLAHE histogram equalization, and at the same time the input images are resized to 600x800 pixels (Fig. 4(b)). Improving image quality helps to find more matching points between the two images. Next, we are looking for specific patterns or specific features, such as corners and edges, which are unique and can be easily tracked and compared. We search and find these features in the first and the second image using the BEBLID descriptor [38], which detects and extracts them. The Brute-Force descriptor matcher [39] is used to compare the two sets of keypoint descriptors and to generate a list of matches. A pair of food images is considered to be relevant if it produces at least 50 matches.

#### 2) Pose Estimation

Camera pose (rotation and translation matrix) is referred to the position and orientation of a camera in relation to the world coordinate system, known as the reference coordinate system. To achieve this, we first filter the inliers from the outliers matching points by calculating the homographic matrix using the RANSAC-based robust method [40]. The RANSAC maximum reprojection error for treating a point as inlier is set to 0.6% of the largest image dimension and the level of confidence is set to 99%. In addition, to further improve the coordinates of corresponding inliers, we use the optimal triangulation method, where for each given inlier correspondence *inliers1[i] ↔ inliers2[i]* and the fundamental matrix F from the corresponding inliers, it computes the corrected correspondences *newInliers1[i] ↔ newInliers2[i]* that minimize the geometric error *d(inliers1[i], newInliers1[i])^2^+d(inliers2[i],newInliers2[i])^2^* (Fig. 4(c)), (where *d(a,b)* is the geometric distance between inliers *a* and *b*), subject to the epipolar constraint newInliers2^T^*F*newInliers1 = 0 (Fig. 4(d)). Finally we can retrieve the relative camera rotation and the translation matrices from the estimated essential matrix and the corresponding inliers in two images, using the cheirality check [41].

#### 3) Scale Determination

In order to estimate the scale factor of the image, a reference card with known dimensions is placed next to the dish. To find the scale factor, we first detect the reference card in the image followed by its 3D reconstruction to obtain the corresponding 3D coordinates. For reference card detection we use a pattern of the card and we compute the homography between the food image and the pattern (Fig. 4(e)). Let *d_Ref_* be the real distance of reference card and *d_Est_* be its size as obtained by estimation in 3D, then the scale factor is computed using the ratio *d_Ref_/d_Est_*.

#### 4) Stereo Matching

To estimate the dense depth map between the two images, first the image pair is rectified, so that we can search for corresponding pixels between them (Fig. 4(f)). Stereo rectification reprojects the images to a new common plane parallel to the line between the camera centers. If the images have been rectified correctly, then a point in the first image and a point in the second image, that correspond to the same 3D world point, will be on the same vertical coordinate. Next, we apply stereo matching techniques to estimate the dense depth map, by finding the disparity map among corresponding pixels in rectified images. To create the disparity map we use a modified version of the algorithm proposed in [42]. Finally, in this step we apply a weighted least square (wls) filter to align the disparity map edges with those of the source image and to propagate the disparity values from high- to low-confidence regions (Fig. 4(g)).

#### 5) 3D Reconstruction and Volume Estimation

To calculate the volume of the food we need to reconstruct its 3D shape, creating the corresponding 3D point cloud. Next, we convert the point cloud to a 3D surface, using the Delaunay triangulation [43], building a triangle mesh over the existing vertices of the point cloud (Fig. 4(h)). Knowing that from a stereo vision method we obtained an unstructured point cloud, we perform surface reconstruction to get a network of triangles. The last step of the proposed volume estimation subsystem is to calculate the amount of the food. To achieve this, we need to extract the food from the surface of the dish. Considering that the reference card defines the table plane and the bottom of the dish, we apply the RANSAC algorithm to find the plane with the largest support in the point cloud (Fig. 4(h)). Then, only the 3D points above this plane are used to estimate the volume of the food computing the convex hull of triangles. Finally, the USDA database can be used to estimate the weight of the food in grams using (1):

\begin{equation*}
{W}_{est} = {V}_{est}*{W}_{USDA }/ {V}_{USDA}, \tag{1}
\end{equation*}

where *V_est_* is the estimated volume of food, while *W_USDA_* and *V_USDA_* are the weight and volume of food, respectively, in the USDA food database. Finally, we use the Greek Food Composition Dataset to calculate macronutrients, such as carbohydrates, proteins, and fats through (2):

\begin{equation*}
Macr{o}_{est} = Macr{o}_{GFCD}*{W}_{est} /100,\ \tag{2}
\end{equation*}

where *Macro_est_* is the estimated macronutrient, while *Macro_GFCD_* is the macronutrient of food in Greek Food Composition Dataset and *W_est_* is the estimated volume of food.

### D. Implementation

We used the python programming language to implement the dietary assessment system in the Anaconda environment. Knowing the increased computing power requirements of CNN models, we also used the cuda toolkit, the cudnn and tensorflow libraries, for model training and validation of classification subsystem, through the Nvidia GeForce RTX 3080 graphic processing unit. Also, we used the opencv, scipy and open3d libraries for the implementation of food volume estimation subsystem.

## III. Results

### A. Food Classification Subsystem

To evaluate the proposed food classification subsystem, we further constructed and trained four well-known CNN classification models. The additional CNN classification models we used and trained are EfficientNetB2, ResNet50 [44], InceptionV3 [45] and DenseNet169 [46]. In all four models, we applied the fine-tuning technique using the pre-trained weights in the ImageNet dataset [47] for the frozen layers. In the proposed model, we applied the fine-tuning using the weights of the pre-trained EfficientNetB2 model in the Food-101 dataset, which is the methodology we propose to evaluate the food classification subsystem. More specifically, in the first model (EfficientNetB2), we applied the fine-tuning technique retraining the last two convolutional blocks of layers in the MedGRFood dataset. By using this model, we achieved 80.6% and 96.7% top-1 and top-5 accuracy, respectively, while the loss of the model was 0.85. In the second model (ResNet50), we applied the fine-tuning technique retraining the last convolutional block of layers in the MedGRFood dataset. By using the second model, we achieved 78.2% and 95.8% top-1 and top-5 accuracy, respectively, while the loss of the model was 1.13. In the third model (InceptionV3), we applied the fine-tuning technique retraining the last two convolutional blocks of layers in the MedGRFood dataset. By using the third model, we achieved 79.3% and 96.1% top-1 and top-5 accuracy, respectively, while the loss of the model was 1.02. In the fourth model (DenseNet169), we applied the fine-tuning technique retraining the last convolutional block of layers in the MedGRFood dataset. By using the fourth model, we achieved 81.1% and 96.5% top-1 and top-5 accuracy, respectively, while the loss of the model was 0.90. In the proposed model, we first trained our model in the Food-101 dataset. Then, we used these weights for the first layers of the base model, while for the last two blocks of layers, we applied the fine-tuning technique, retraining them in the MedGRFood dataset. The top-1 and top-5 accuracy for the proposed method were 83.8% and 97.6%, respectively, while the loss was 0.68. Table I presents the classification results of the five models. All models have been trained for 250 epochs using the same hardware. The MedGRFood dataset is partitioned into the training and validation set, using a ratio of 78:22 (78% is used for model training and 22% is used for model validation).

**TABLE I:** Classification Results Between the Five Models

Model	Top-1 Accuracy (%)	Top-5 Accuracy (%)	Loss	Recall	F1-score
First model (EfficientNetB2)	80.6	96.7	0.85	0.81	0.80
Second model (ResNet50)	78.2	95.8	1.13	0.78	0.78
Third model (InceptionV3)	79.3	96.1	1.02	0.79	0.79
Fourth model (DenseNet169)	81.1	96.5	0.90	0.81	0.81
Proposed model	**83.8**	**97.6**	**0.68**	**0.84**	**0.83**

**Fig. 5.**
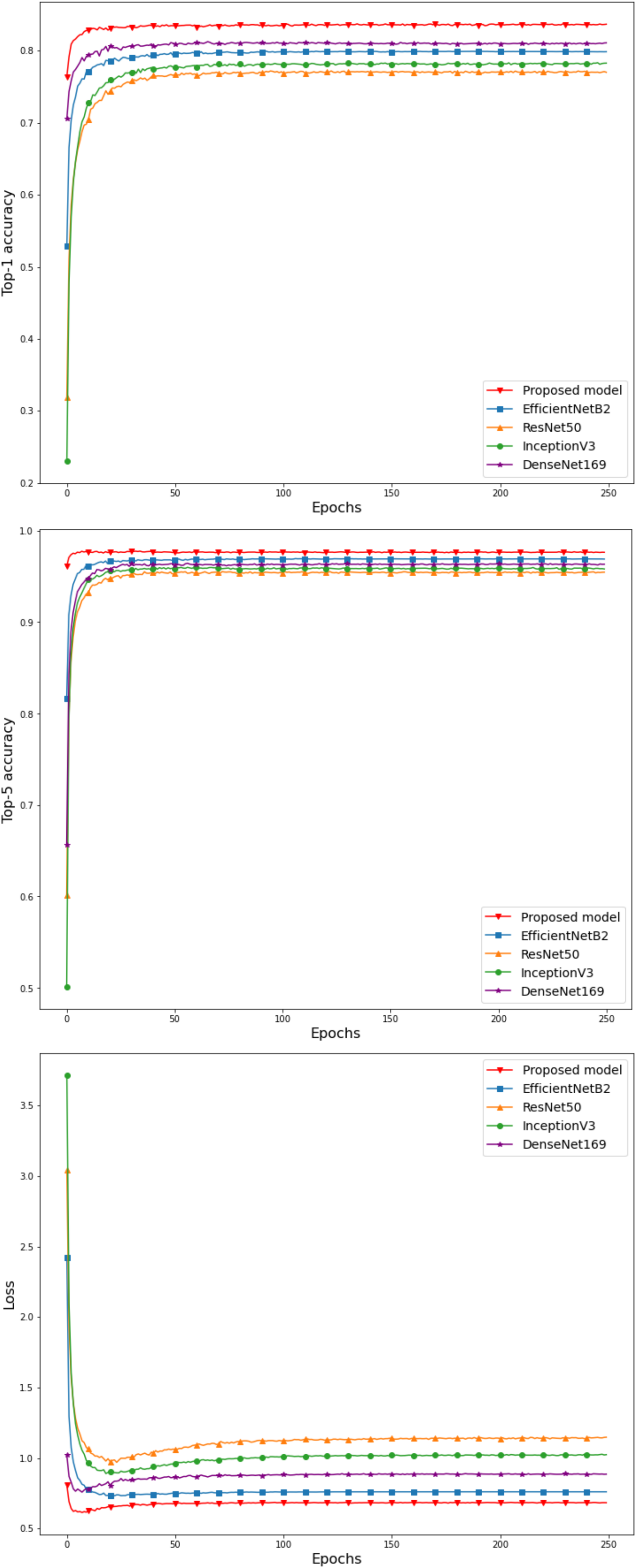
Top-1, top-5 and loss comparison between the classification models.

We observe that the first four models which are pre-trained in the ImageNet dataset, have lower values in the top-1, top -5, recall and f1-score indices while they have a higher value in the loss index. The comparison of the classification models with the proposed model shows that the use of pre-trained weights from a relevant dataset with the dataset of images we want to classify, leads to obtaining better results than training the classification model using pre-trained weights of a more general image dataset. In addition, it is worth mentioning the significant difference in the loss index, between these models, where our proposed methodology achieves the lowest value. Fig. 5 shows the curves of the top-1 accuracy, top-5 accuracy and loss indices in the validation set between the five classification models, proving the efficiency of the proposed model for food image classification. Overall, we see that the proposed model has the best values in all indices, followed by EfficientNetB2 along with DenseNet169, InceptionV3 being the fourth in a row in performance and finally ResNet50. Examining the loss index curves, we notice that in CNN ResNet50, InceptionV3 and DenseNet169 networks there is a slight tendency for the index to increase over the epochs, while in the proposed model and in EfficientNetB2 the value of the loss index remains constant. In addition, the proposed model has a very low value for the loss index, that shows how well our model behaves after each iteration of optimization. Also, the comparison between the curves of the models shows that our methodology allows the fastest convergence to the optimal value of each index. The total number of training parameters created between the models is huge, requiring a lot of training time and an increased computational cost and power. In addition, we must not forget that the proposed methodology presupposes the training of the base model in the Food-101 dataset, requiring additional training time. However, the improvement of the top-1 and top-5 accuracy indices and the reduction of the loss index value, make the deep learning model we proposed a better choice to deal with the food classification problems. The high value of the top-1 accuracy index of the proposed methodology, considered that there are several foods in the MedGRFood dataset that may look-alike (i.e., moussaka, pastitsio, lasagna, spinach pie, cheese pie, etc.), make the classification of food difficult and they maintain the top-1 accuracy in a constant value (about 84%). The fact that several foods look similar is evident from the value of the top-5 accuracy index, which is very high and is one of the best top-5 values according to the literature [48]. By achieving better performance with the proposed model pre-trained in food imaging data when compared to the pre-trained models in non-food imaging data, we proved the superiority of the proposed classification model.

### B. Food Volume Estimation Subsystem

To evaluate the food volume, we have calculated the mean absolute percentage error (MAPE) for the food dishes, and have made five different estimates for each dish from the MedGRFood dataset. Equation (3) is used to calculate *MAPE_i_* of each dish [18].

\begin{equation*}
MAP{E}_i = \frac{1}{5}\sum_{j = 1}^5 \left| {\frac{{{V}_{real} - {V}_{est}}}{{{V}_{real}}}} \right|, \tag{3}
\end{equation*}

where *V_real_* is the real volume of food, while *V_est_* is the estimated volume. To calculate the overall MAPE of the food volume estimation subsystem we use (4). In total, we estimate the volume of 148 dishes from the MedGRFood image dataset.

\begin{equation*}
MAP{E}_{overall} = \frac{1}{{148}}\sum_{i = 1}^{148} MAP{E}_i, \tag{4}
\end{equation*}

**Fig. 6.**
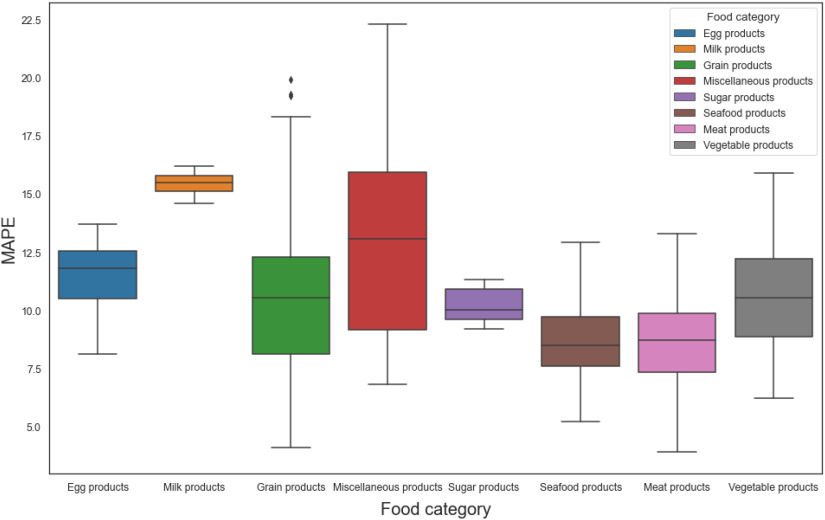
MAPE distribution for each food category.

The overall MAPE of the proposed food volume estimation subsystem is 10.5%. Table II shows the number of different dishes and the MAPE index for each food category from the MedGRFood dataset. We observe that categories of foods that contain dishes with few and weak features, such as milk products, present a higher MAPE compared to categories of dishes with many and strong features, such as meat products. In Fig. 6 we observe that the dishes belonging to the miscellaneous products category have the largest distribution, which is explained by the diversity of dishes belonging to this category. Finally, categories such as seafood, meat and vegetables products a controlled distribution as they consist of dishes with similar features.

**TABLE II:** Subsystem MAPE for Each Food Category

Food category	Number of dishes	MAPE (%)
Milk, dairy products or milk substitutes	1	15.4
Egg or egg products	3	11.4
Meat or meat products	23	8.6
Seafood or related products	20	8.7
Grain or grain products	48	10.3
Vegetable or vegetable products	28	10.6
Sugar or sugar products	1	10.2
Miscellaneous food products	24	13

## IV. Discussion and Conclusion

In this paper we presented an automated image-based dietary assessment system which consists of three major parts: (i) the MedGRFood image dataset, (ii) the food classification subsystem and (iii) the food volume estimation subsystem.

The use of deep learning techniques to classify food images requires the development of datasets with a big amount of imaging data. However, the existing food image datasets are limited to the number of food classes, depending on the dataset constructor's eating habits and the type of cuisine one wants to cover. In addition, image quality has positive or negative effect on the performance of classification systems, so it is equally important that the food image dataset contains high-resolution images. High quality images allow the classification model to be trained with higher-resolution images, in which more details of the food are discernible, creating models having better accuracy. The MedGRFood image dataset is a first step towards the creation of a complete dataset of food images representing the Mediterranean cuisine and diet, according to the Greek Food Composition Dataset by the Hellenic Health Foundation. Expanding the dataset by adding images from missing foods classes and categories, such as fruits, is the next step in completing it, which will provide a comprehensive food dataset with high-resolution images available to the scientific community for research purposes.

By using the EfficientNetB2 pre-trained in Food-101 image dataset as base classification model and by applying fine tuning, transfer learning and data augmentation techniques, we were able to achieve 83.8% top-1 accuracy and 97.6% top-5 accuracy in the MedGRFood food image dataset. By achieving better performance with the proposed model in the Food-101 when compared to the pre-trained models in the ImageNet, we proved the significancy of pre-trained classification model in a relevant dataset. Our methodology presupposes the identification of the appropriate dataset and the training of the base model in the Food-101 dataset. While most food classification models are either trained in a dataset from scratch or use a pre-trained model in the ImageNet dataset, we used a pre-trained model on a food dataset. Therefore, the proposed methodology for image classification, can also be used to classify images from different datasets. Knowing that basic image processing tasks (such as edge detection) are performed in the first layers of a CNN classification model, we used the pre-trained weights of a related dataset to significantly improve all classification indices. We chose the EfficientNet as base model because it achieves both higher accuracy and better efficiency compared to previous CNNs image classification models. Furthermore, the computational cost, the parameters size in addition to the time required to train the model based on EfficientNet are substantially smaller.

Finally, we described and analyzed the procedure for estimating the volume of the food using a stereo vision approach. The proposed volume estimation subsystem presupposes two captured images to calculate the amount of food contained in a shallow dish. The volume estimation pipeline involves 3D reconstruction of food using stereo vision techniques, such as feature detection and extraction, feature matching, pose estimation, scale determination, stereo matching and point cloud generation. The overall MAPE of the proposed methodology is 10.5% for 148 different dishes from the MedGRFood image dataset. The dish with the lowest MAPE is the beefsteak while the dish with the highest MAPE is the fish roe dip, with MAPE values 5.1% and 21.3%, respectively.

Comparing our proposed dietary assessment system to the related literature, we observe that we have created a unique Mediterranean image dataset suitable for both food image classification systems and food volume estimation systems. Moreover, the proposed food classification methodology gives a very good top-1 accuracy, an excellent top-5 accuracy, and a very low loss index. By counting the number of food classes we want to classify and by comparing the classification results with other food image classification models, we observe that our results are among the best in literature. Finally, the proposed volume estimation subsystem calculates the MAPE for the largest number of dishes in the relevant literature.

The main advantage of the proposed methodology is that it is a fully automated process to calculate the nutritional composition capturing food images through a smartphone camera. This is what today is used and it is easy to be used by most of the people and of all ages to capture photos and more specifically food images, which provide the capability of continuous recording of health data in real time. The use of mobile devices and cloud technology to monitor health data and sharing it with physicians, can lead to faster and lower misdiagnosis of diseases, such as diabetes, obesity and CVDs. By creating a new food image dataset and using methods and techniques from computer vision and image processing, we offer a possible solution to the problem of calculating food nutrition through images. A limitation of the proposed methodology is that it estimates the nutritional composition of foods contained only in shallow dishes which must be improved to include different types of dishes (i.e., deep dishes).

## V. Supplementary Materials

The Supplementary Materials includes food images of the MedGRFood dataset, learning rate schedule, higher resolution food point cloud reconstruction images and tables that contain the top-5 dishes with higher and lower MAPE index.

Supplementary materials
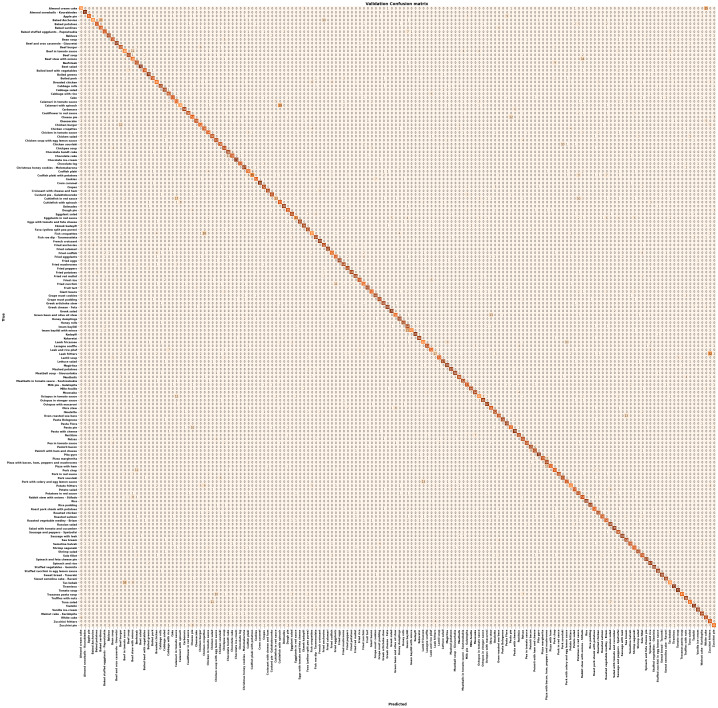

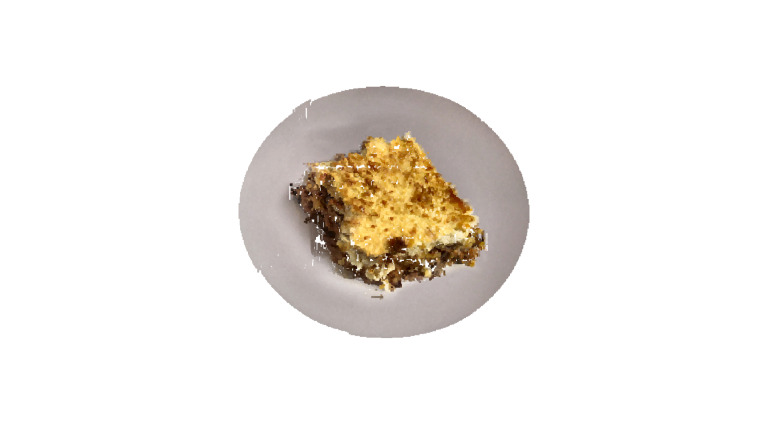

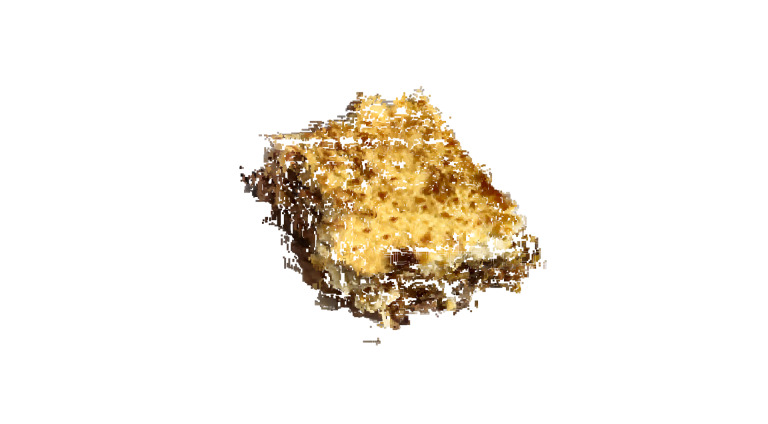

## References

[1] World Health Organization, “Diabetes,” 2022. Accessed: Sep. 16, 2022. [Online]. Available: https://www.who.int/news-room/fact-sheets/detail/diabetes

[2] International Diabetes Federation, “Diabetes facts & figures,” 2022. Accessed: Dec. 19, 2022. [Online]. Available: https://www.idf.org/aboutdiabetes/what-is-diabetes/facts-figures.html

[3] World Health Organization, “Cardiovascular diseases (CVDs),” 2021. Accessed: Jun. 11, 2021. [Online]. Available: https://www.who.int/news-room/fact-sheets/detail/cardiovascular-diseases-(cvds)

[4] World Health Organization, “Obesity and overweight,” 2021. Accessed: Jun. 9, 2021. [Online]. Available: https://www.who.int/news-room/fact-sheets/detail/obesity-and-overweight

[5] C. Koliaki, S. Liatis, and A. Kokkinos, “Obesity and cardiovascular disease: Revisiting an old relationship,” Metabolism, vol. 92, pp. 98–107, 2019. 30399375 10.1016/j.metabol.2018.10.011

[6] T. P. Velavan and C. G. Meyer, “The COVID-19 epidemic,” Trop. Med. Int. Health, vol. 25, no. 3, pp. 278–280, 2020. 32052514 10.1111/tmi.13383 PMC7169770

[7] World Health Organization, “Coronavirus disease (COVID-19),” 2021. Accessed: May 12, 2021. [Online]. Available: https://www.who.int/emergencies/diseases/novel-coronavirus-2019

[8] Y. Zheng, S. H. Ley, and F. B. Hu, “Global aetiology and epidemiology of type 2 diabetes mellitus and its complications,” Nature Rev. Endocrinol., vol. 14, no. 2, pp. 88–98, 2018. 29219149 10.1038/nrendo.2017.151

[9] J. Sharifi-Rad , “Diet, lifestyle and cardiovascular diseases: Linking pathophysiology to cardioprotective effects of natural bioactive compounds,” Int. J. Environ. Res. Public Health, vol. 17, no. 7, 2020, Art. no. 2326. 10.3390/ijerph17072326 PMC7177934 32235611

[10] S. Fang, Z. Shao, D. A. Kerr, C. J. Boushey, and F. Zhu, “An end-to-end image-based automatic food energy estimation technique based on learned energy distribution images: Protocol and methodology,” Nutrients, vol. 11, no. 4, 2019, Art. no. 877. 10.3390/nu11040877 PMC6521161 31003547

[11] L. Bossard, M. Guillaumin, and L. Van Gool, “Food-101–mining discriminative components with random forests,” in Proc. Eur. Conf. Comput. Vis., 2014, pp. 446–461.

[12] J. Chen and C.-W. Ngo, “Deep-based ingredient recognition for cooking recipe retrieval,” in Proc. 24th Assoc. Comput. Machinery Int. Conf. Multimedia, 2016, pp. 32–41.

[13] Y. Matsuda, H. Hoashi, and K. Yanai, “Recognition of multiple-food images by detecting candidate regions,” in Proc. IEEE Int. Conf. Multimedia Expo., 2012, pp. 25–30.

[14] G. Ciocca, P. Napoletano, and R. Schettini, “Food recognition: A new dataset, experiments, and results,” IEEE J. Biomed. Health Inform., vol. 21, no. 3, pp. 588–598, May 2017. 28114043 10.1109/JBHI.2016.2636441

[15] Y. Kawano and K. Yanai, “Automatic expansion of a food image dataset leveraging existing categories with domain adaptation,” in Proc. Eur. Conf. Comput. Vis., 2014, pp. 3–17.

[16] M. M. Anthimopoulos, L. Gianola, L. Scarnato, P. Diem, and S. G. Mougiakakou, “A food recognition system for diabetic patients based on an optimized bag-of-features model,” IEEE J. Biomed. Health Inform., vol. 18, no. 4, pp. 1261–1271, Jul. 2014. 25014934 10.1109/JBHI.2014.2308928

[17] L. Xiao, T. Lan, D. Xu, W. Gao, and C. Li, “A simplified CNNs visual perception learning network algorithm for foods recognition,” Comput. Elect. Eng., vol. 92, 2021, Art. no. 107152.

[18] J. Dehais, M. Anthimopoulos, S. Shevchik, and S. Mougiakakou, “Two-view 3D reconstruction for food volume estimation,” IEEE Trans. Multimedia, vol. 19, pp. 1090–1099, 2017.

[19] Y. Ando, T. Ege, J. Cho, and K. Yanai, “Depthcaloriecam: A mobile application for volume-based foodcalorie estimation using depth cameras,” in Proc. 5th Int. Workshop Multimedia Assist. Dietary Manage., 2019, pp. 76–81.

[20] W. Jia , “Accuracy of food portion size estimation from digital pictures acquired by a chest-worn camera,” Public Health Nutr., vol. 17, no. 8, pp. 1671–1681, 2014. 24476848 10.1017/S1368980013003236 PMC4152011

[21] P. Pouladzadeh, S. Shirmohammadi, and R. Al-Maghrabi, “Measuring calorie and nutrition from food image,” IEEE Trans. Instrum. Meas., vol. 63, no. 8, pp. 1947–1956, Aug. 2014.

[22] Q. Thames , “Nutrition5k: Towards automatic nutritional understanding of generic food,” in Proc. IEEE/CVF Conf. Comput. Vis. Pattern Recognit., 2021, pp. 8903–8911.

[23] G. A. Tahir and C. K. Loo, “A comprehensive survey of image-based food recognition and volume estimation methods for dietary assessment,” Healthcare, vol. 9, no. 12, 2021, Art. no. 1676. 10.3390/healthcare9121676 PMC8700885 34946400

[24] A. Trichopoulou and K. Georga, “Composition tables of foods and Greek dishes,” School of Medicine, 2004.

[25] F. S. Konstantakopoulos, E. I. Georga, and D. I. Fotiadis, “Mediterranean food image recognition using deep convolutional networks,” in Proc. IEEE 43rd Annu. Int. Conf. Eng. Med. Biol. Soc., 2021, pp. 1740–1743. 10.1109/EMBC46164.2021.9630481 34891623

[26] F. Konstantakopoulos, E. I. Georga, and D. I. Fotiadis, “3D Reconstruction and volume estimation of food using stereo vision techniques,” in Proc. IEEE 21st Int. Conf. Bioinf. Bioeng., 2021, pp. 1–4.

[27] F. S. Konstantakopoulos , “GlucoseML mobile application for automated dietary assessment of mediterranean food,” in Proc. IEEE 44th Annu. Int. Conf. Eng. Med. Biol. Soc., 2022, pp. 1432–1435. 10.1109/EMBC48229.2022.9871732 36085710

[28] S. Gebhardt , “USDA national nutrient database for standard reference, release 21,” United States Dept. Agriculture, Agricultural Res. Serv., 2006.

[29] A. Trichopoulou , “Definitions and potential health benefits of the Mediterranean diet: Views from experts around the world,” Biomed. Central Med., vol. 12, no. 1, 2014, Art. no. 112. 10.1186/1741-7015-12-112 PMC4222885 25055810

[30] V. Tosti, B. Bertozzi, and L. Fontana, “Health benefits of the Mediterranean diet: Metabolic and molecular mechanisms,” J. Gerontol.: Ser. A, vol. 73, no. 3, pp. 318–326, 2018. 10.1093/gerona/glx227 PMC7190876 29244059

[31] J. Salas-Salvadó, N. Becerra-Tomás, J. F. García-Gavilán, M. Bulló, and L. Barrubés, “Mediterranean diet and cardiovascular disease prevention: What do we know?,” Prog. Cardiovasc. Dis., vol. 61, no. 1, pp. 62–67, 2018. 29678447 10.1016/j.pcad.2018.04.006

[32] M. Tan and Q. Le, “EfficientNet: Rethinking model scaling for convolutional neural networks,” in Proc. Int. Conf. Mach. Learn., 2019, pp. 6105–6114.

[33] M. Sandler, A. Howard, M. Zhu, A. Zhmoginov, and L.-C. Chen, “Mobilenetv2: Inverted residuals and linear bottlenecks,” in Proc. IEEE Conf. Comput. Vis. Pattern Recognit., 2018, pp. 4510–4520.

[34] L. Bottou, “Large-scale machine learning with stochastic gradient descent,” in Proc. 19th COMPSTAT Int. Conf. Comput. Statist., 2010, pp. 177–186.

[35] X. Zhang, D. Chang, W. Qi, and Z. Zhan, “A Study on different functionalities and performances among different activation functions across different ANNs for image classification,” J. Phys.: Conf. Ser., vol. 1732, no. 1, 2021, Art. no. 012026.

[36] W. Rawat and Z. Wang, “Deep convolutional neural networks for image classification: A comprehensive review,” Neural Comput., vol. 29, no. 9, pp. 2352–2449, 2017. 28599112 10.1162/NECO_a_00990

[37] J. Wang and L. Perez, “The effectiveness of data augmentation in image classification using deep learning,” Convolutional Neural Netw. Vis. Recognit., vol. 11, pp. 1–8, 2017.

[38] I. Suárez, G. Sfeir, J. M. Buenaposada, and L. Baumela, “BEBLID: Boosted efficient binary local image descriptor,” Pattern Recognit. Lett., vol. 133, pp. 366–372, 2020.

[39] F. K. Noble, “Comparison of OpenCV's feature detectors and feature matchers,” in Proc. IEEE 23rd Int. Conf. Mechatronics Mach. Vis. Pract., 2016, pp. 1–6.

[40] R. Raguram, J.-M. Frahm, and M. Pollefeys, “A comparative analysis of RANSAC techniques leading to adaptive real-time random sample consensus,” in Proc. 10th Eur. Conf. Comput. Vis., 2008, pp. 500–513.

[41] D. Nistér, “An efficient solution to the five-point relative pose problem,” IEEE Trans. Pattern Anal. Mach. Intell., vol. 26, no. 6, pp. 756–770, Jun. 2004. 18579936 10.1109/TPAMI.2004.17

[42] H. Hirschmuller, “Stereo processing by semiglobal matching and mutual information,” IEEE Trans. Pattern Anal. Mach. Intell., vol. 30, no. 2, pp. 328–341, Feb. 2008. 18084062 10.1109/TPAMI.2007.1166

[43] Y. Xu, K. Liu, J. Ni, and Q. Li, “3D reconstruction method based on second-order semiglobal stereo matching and fast point positioning delaunay triangulation,” PLoS One, vol. 17, no. 1, 2022, Art. no. e0260466. 10.1371/journal.pone.0260466 PMC8789135 35077460

[44] K. He, X. Zhang, S. Ren, and J. Sun, “Deep residual learning for image recognition,” in Proc. IEEE Conf. Comput. Vis. Pattern Recognit., 2016, pp. 770–778.

[45] C. Szegedy, V. Vanhoucke, S. Ioffe, J. Shlens, and Z. Wojna, “Rethinking the inception architecture for computer vision,” in Proc. IEEE Conf. Comput. Vis. Pattern Recognit., 2016, pp. 2818–2826.

[46] G. Huang, Z. Liu, L. Van Der Maaten, and K. Q. Weinberger, “Densely connected convolutional networks,” in Proc. IEEE Conf. Comput. Vis. Pattern Recognit., 2017, pp. 4700–4708.

[47] A. Krizhevsky and I. Sutskever, and G. E. Hinton, “Imagenet classification with deep convolutional neural networks,” in *Proc*. Adv. Neural Inf. Process. Syst., 2012, pp. 1097–1105.

[48] F. P. W. Lo, Y. Sun, J. Qiu, and B. Lo, “Image-based food classification and volume estimation for dietary assessment: A review,” IEEE J. Biomed. Health Inform., vol. 24, no. 7, pp. 1926–1939, Jul. 2020. 32365038 10.1109/JBHI.2020.2987943

