# Supplementary material for: An Automated Image-Based Dietary Assessment System for Mediterranean Foods
Source: IEEE Open J Eng Med Biol. 2023 Apr 13;4:45–54. doi: 10.1109/OJEMB.2023.3266135 (PMC10202193; doi:10.1109/OJEMB.2023.3266135)
Supplement: Supplementary materials [file supp1-3266135.docx]

**Supplementary Materials**

An Automated Image-Based Dietary Assessment System for Mediterranean Foods

Fotios S. Konstantakopoulos, Eleni I. Georga, and Dimitrios I. Fotiadis, Fellow, IEEE

| 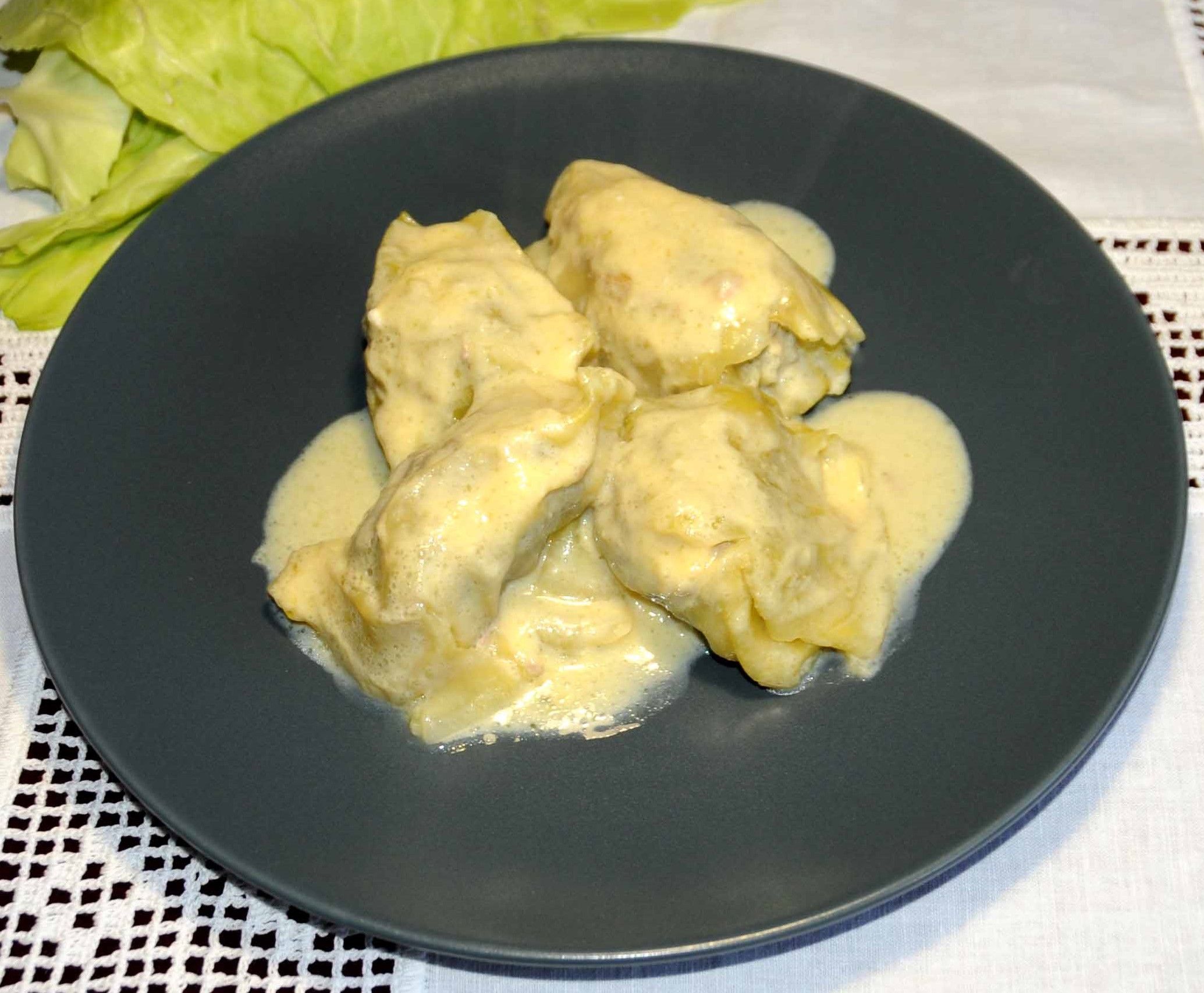 | 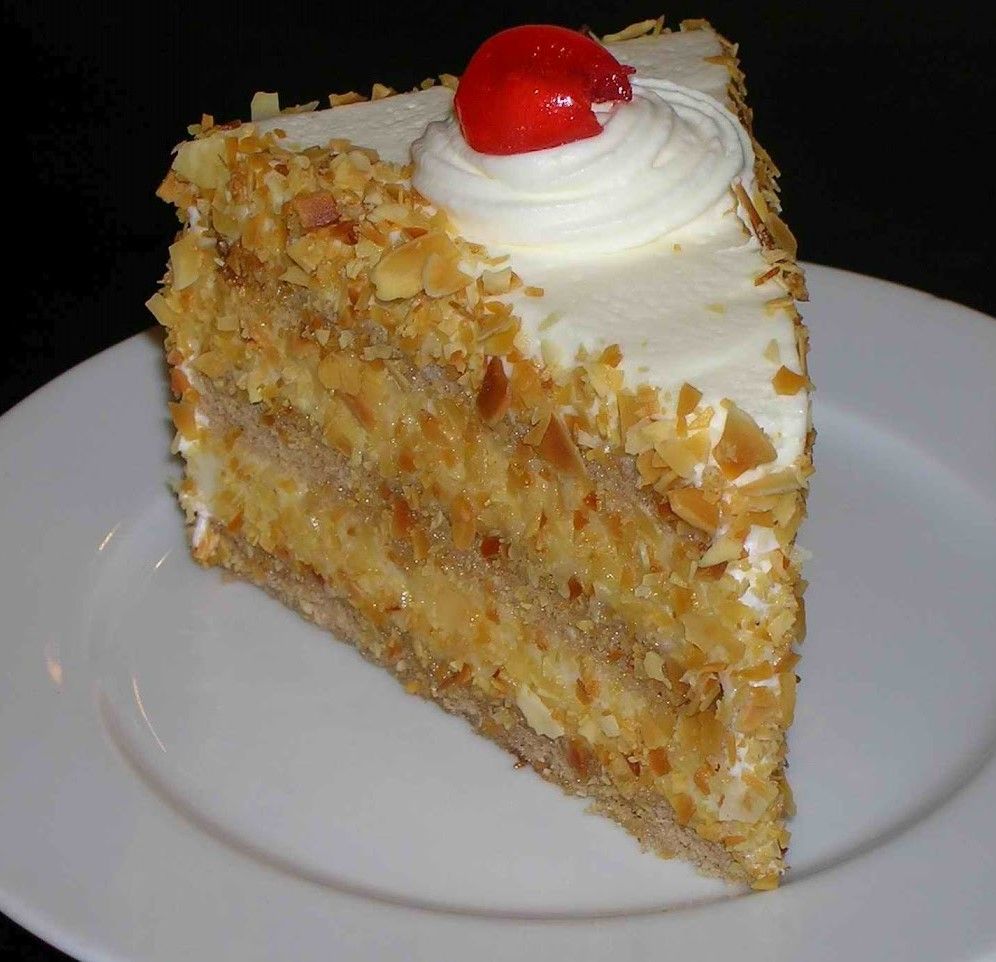 | 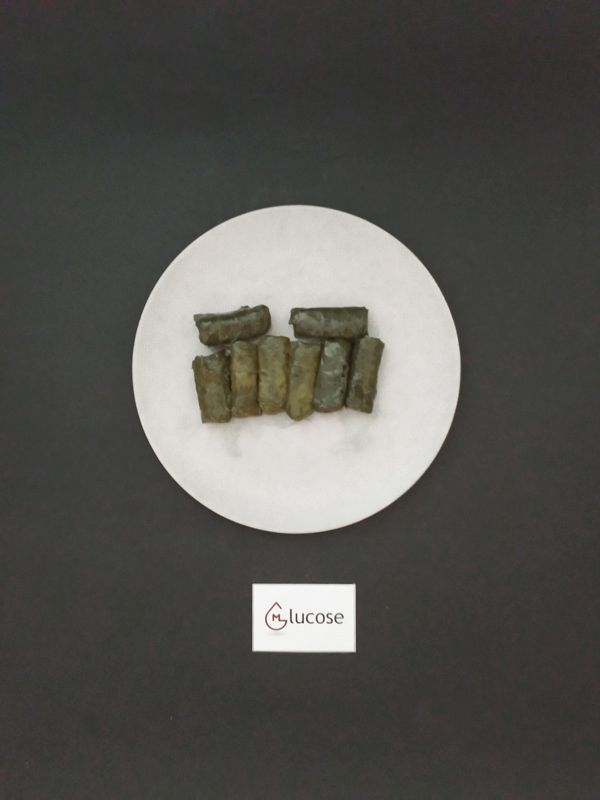 | 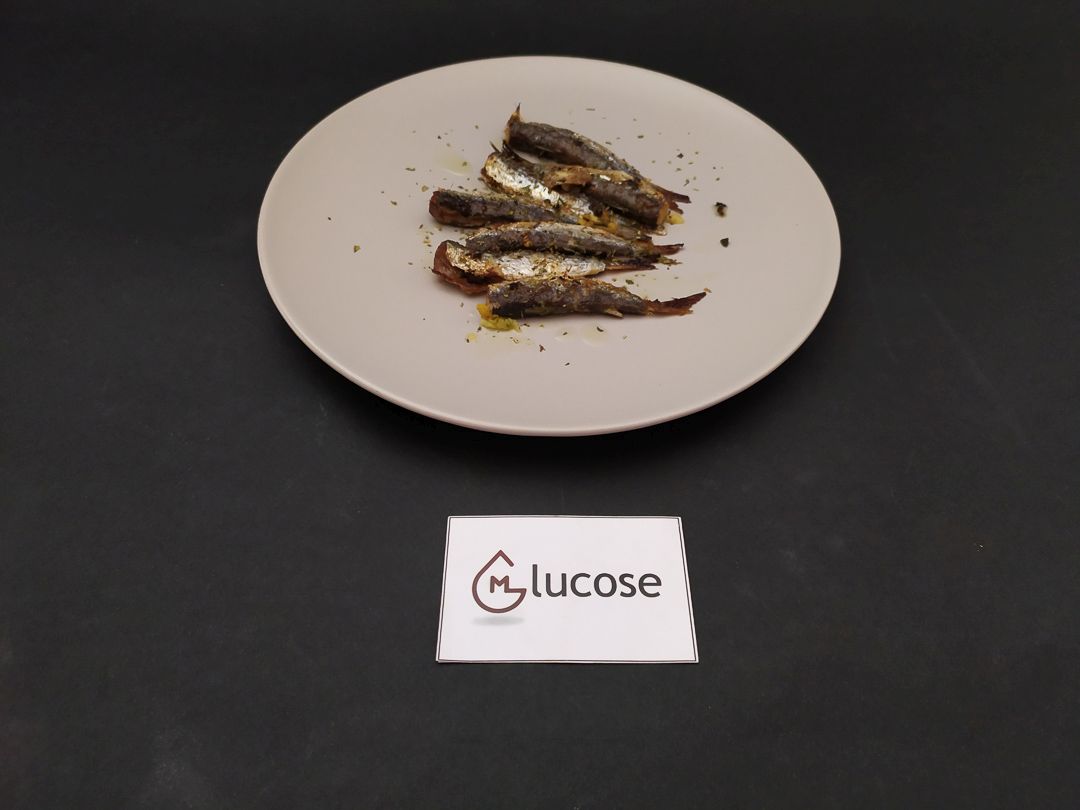 | 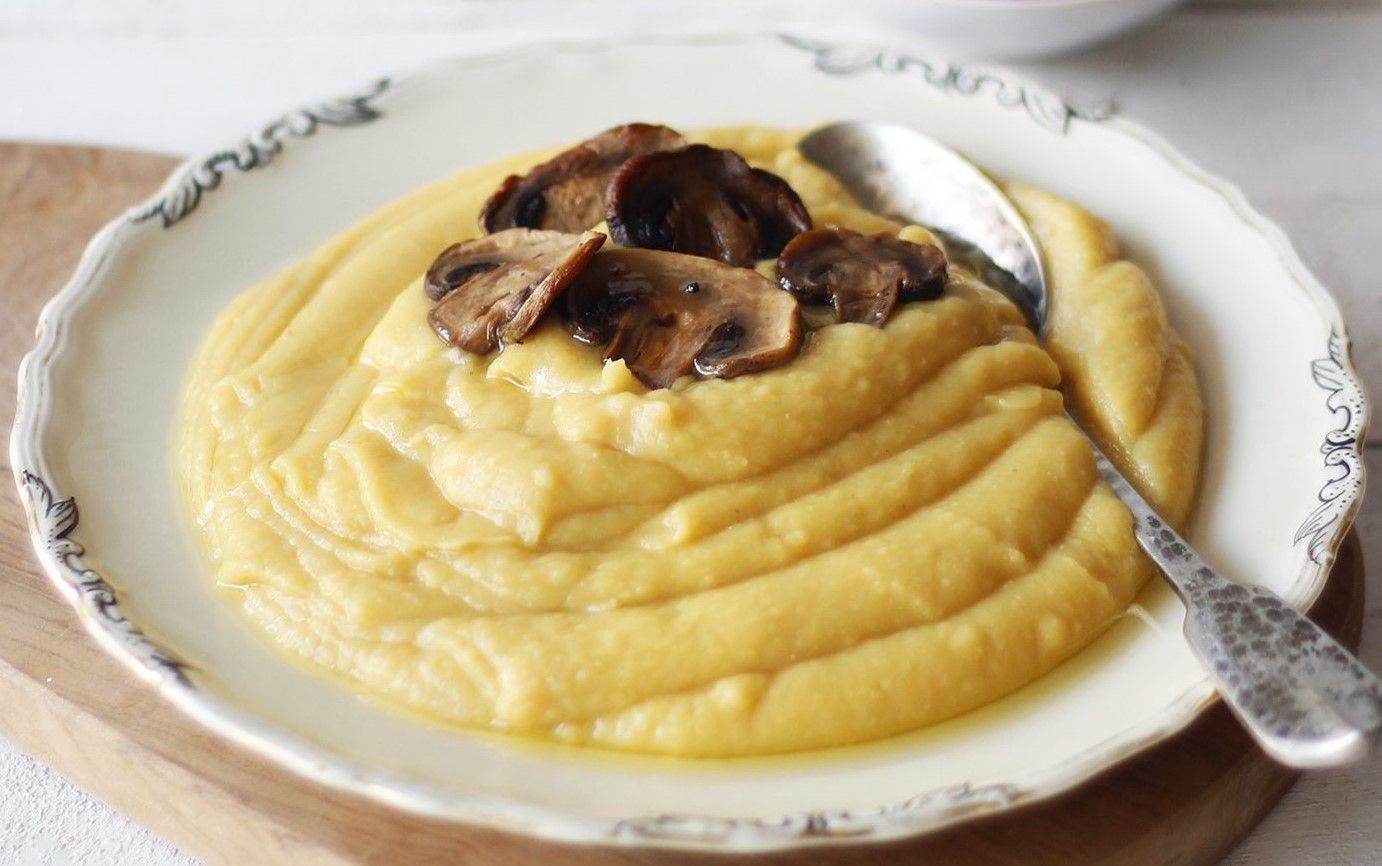 | 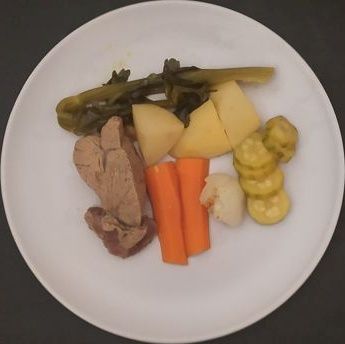 | 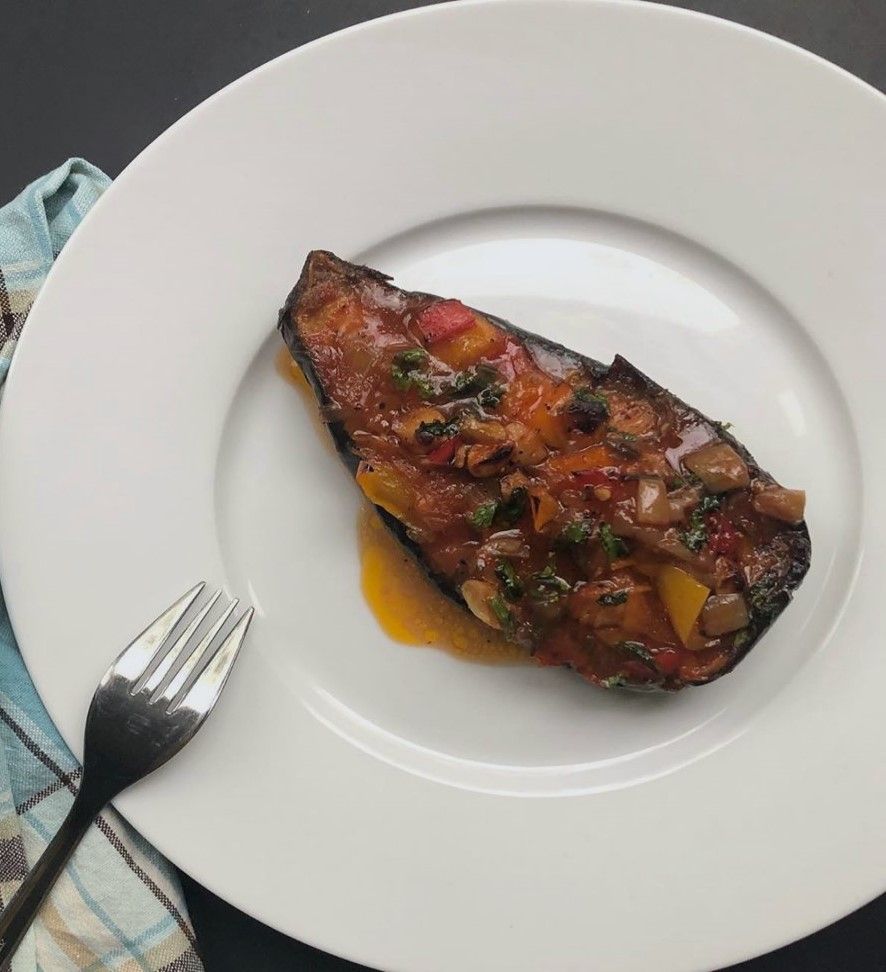 | 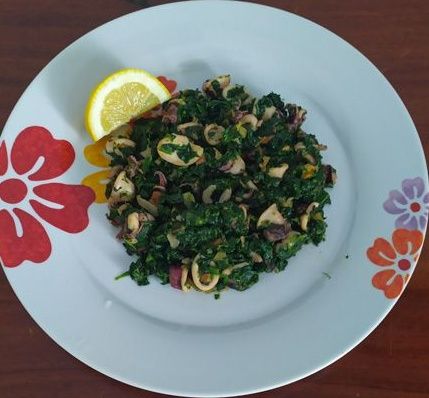 |
| --- | --- | --- | --- | --- | --- | --- | --- |
| 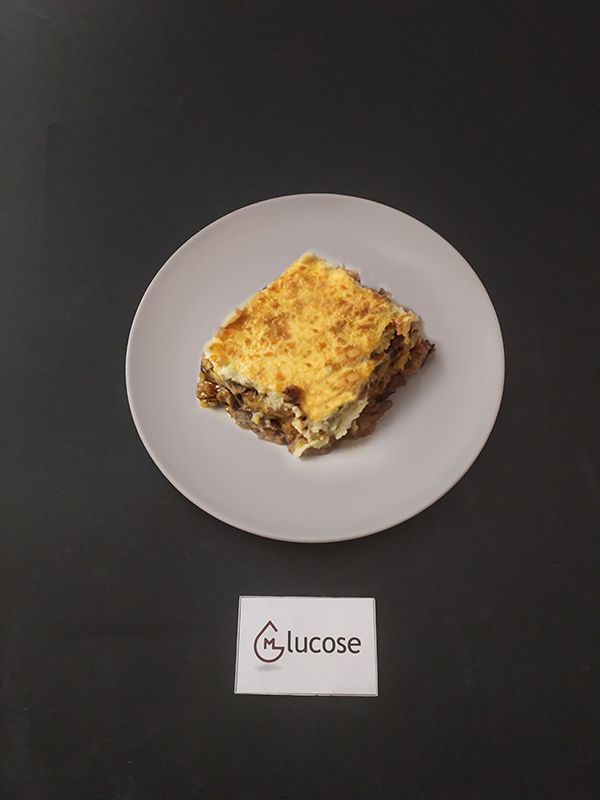 | 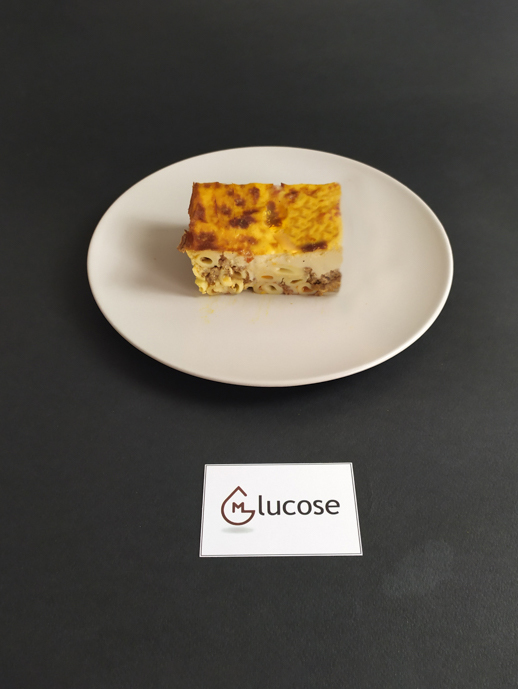 | 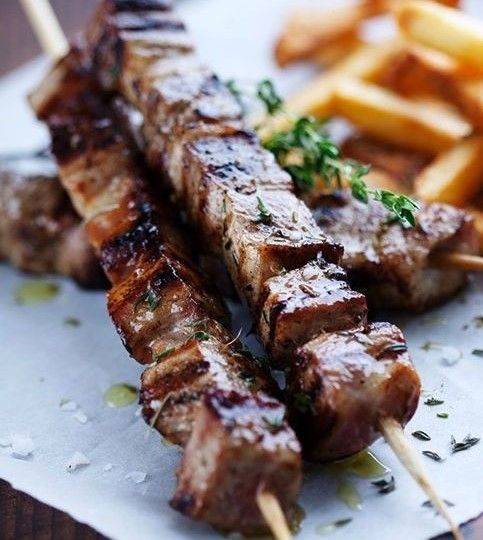 | 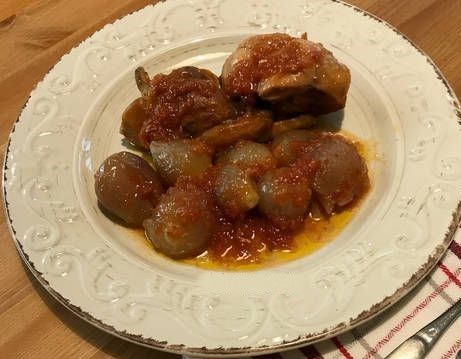 | 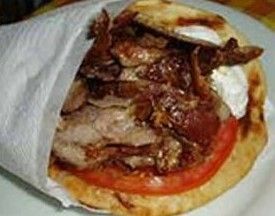 | 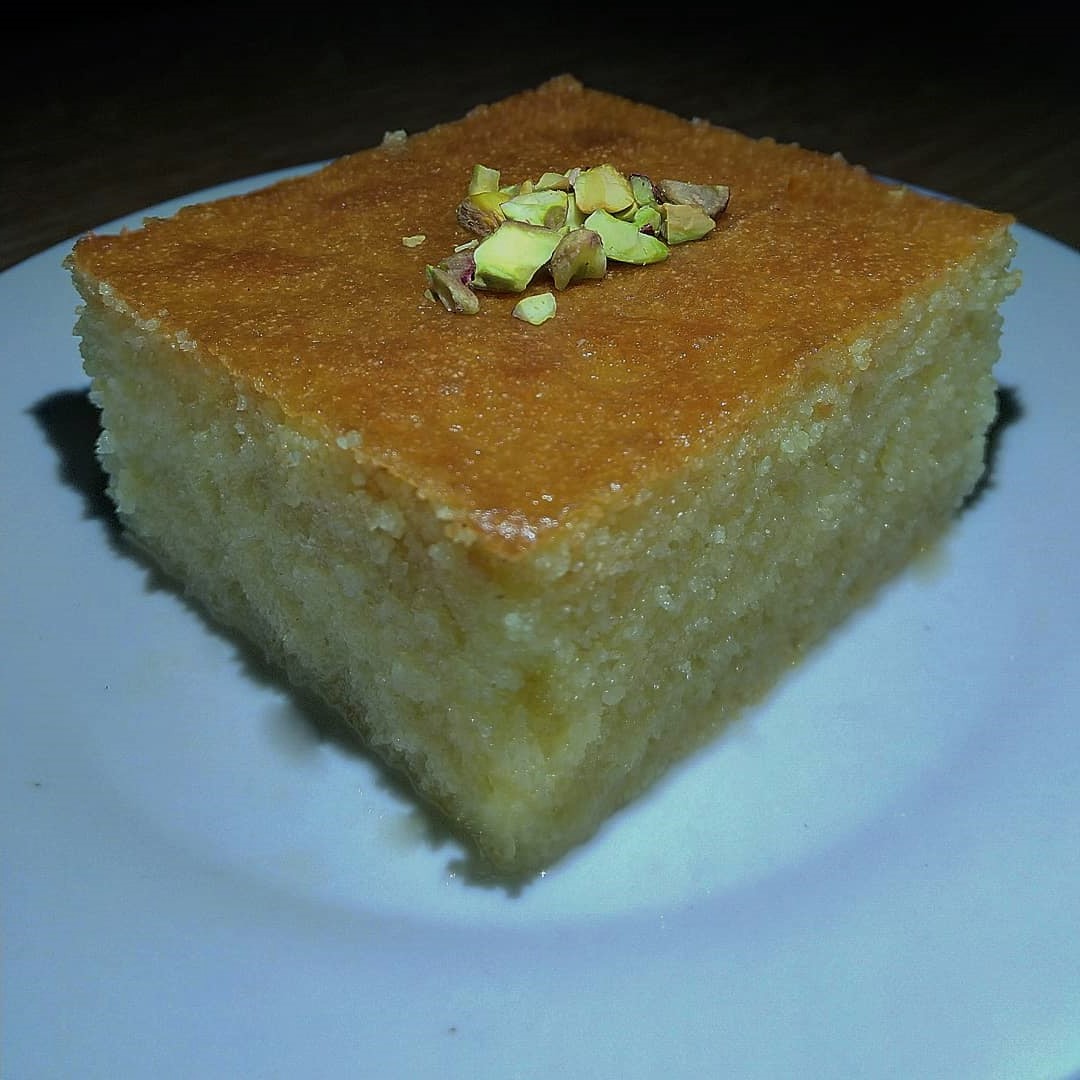 | 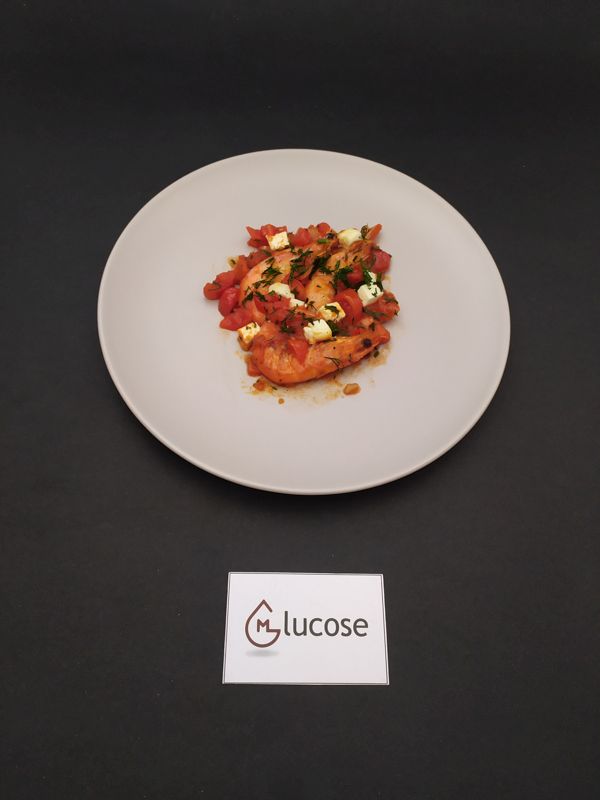 | 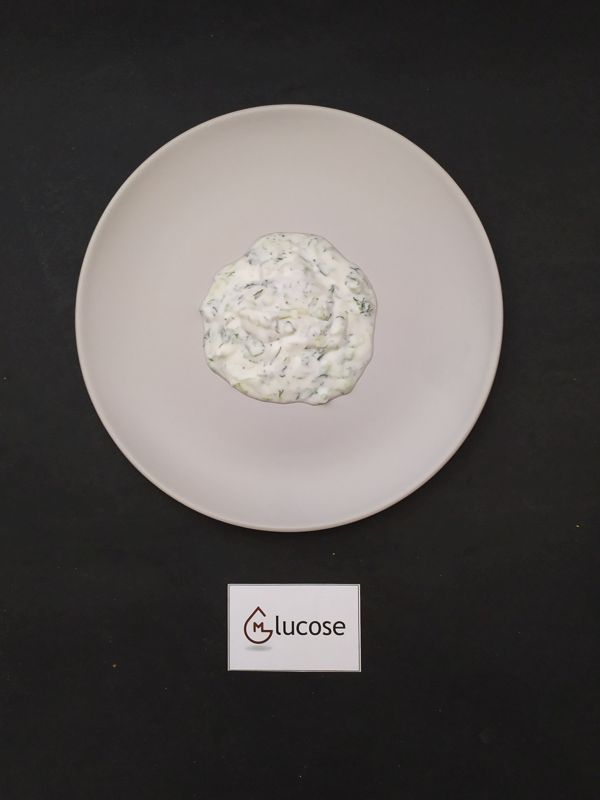 |
| 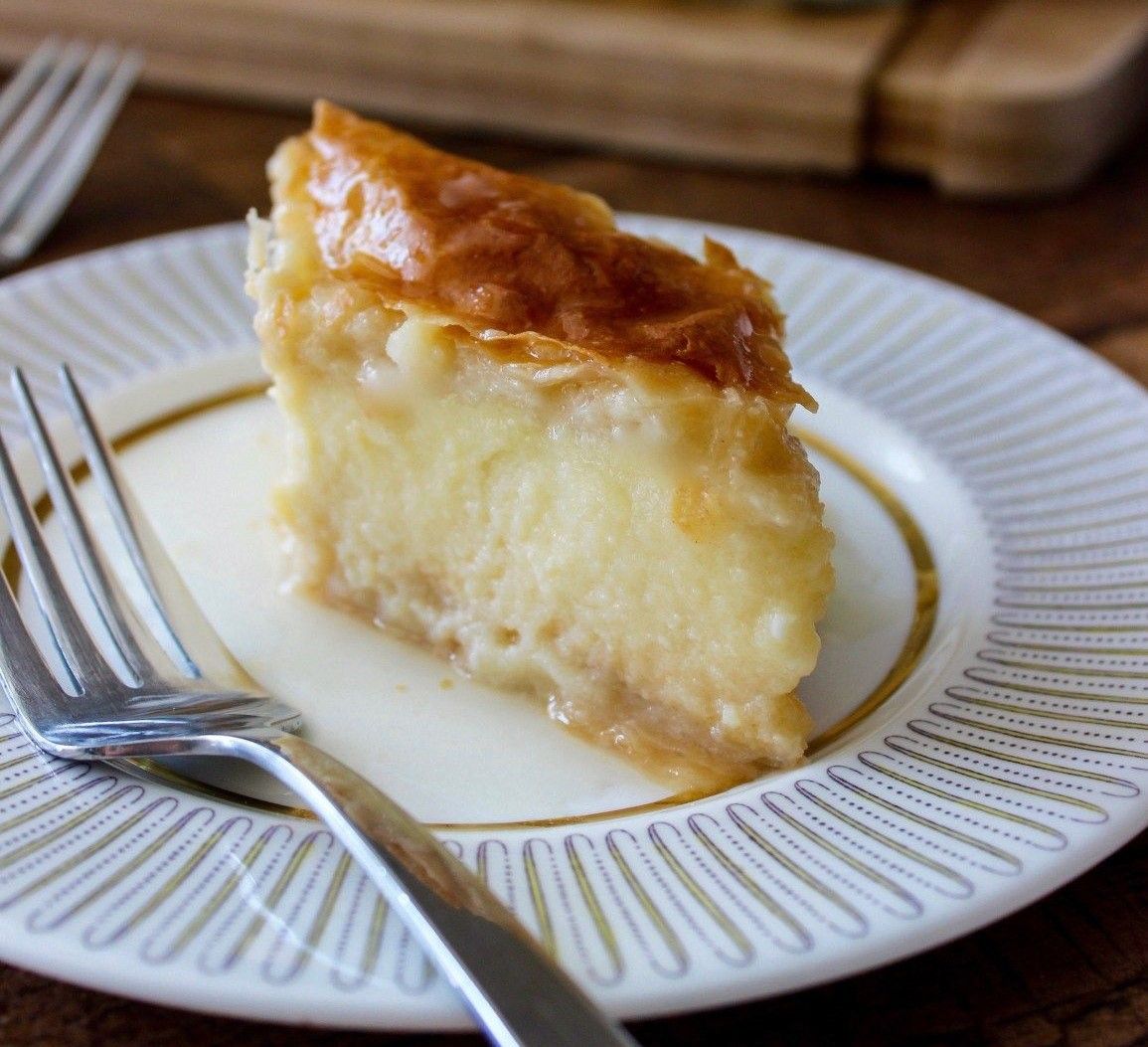 | 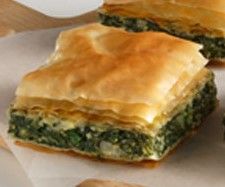 | 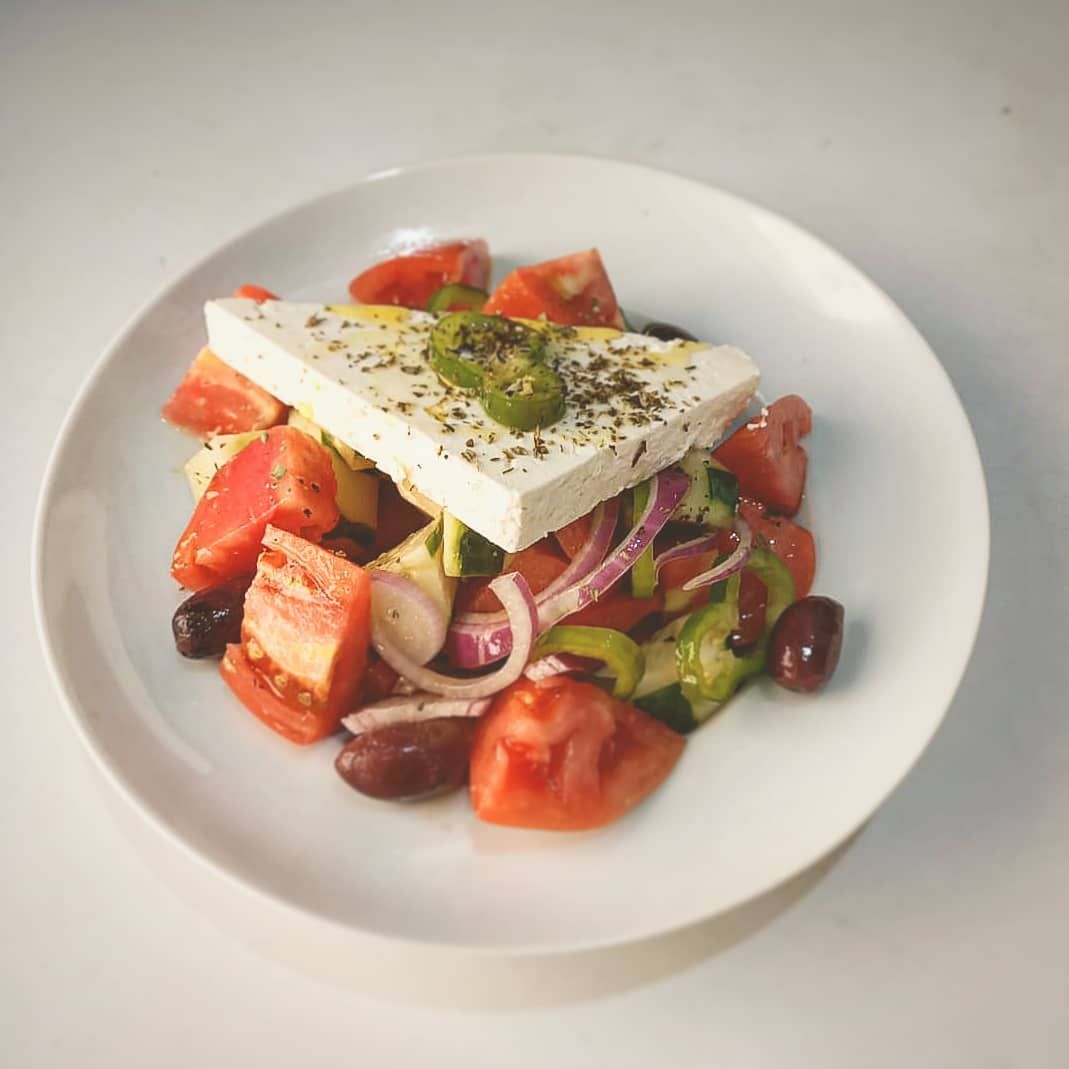 | 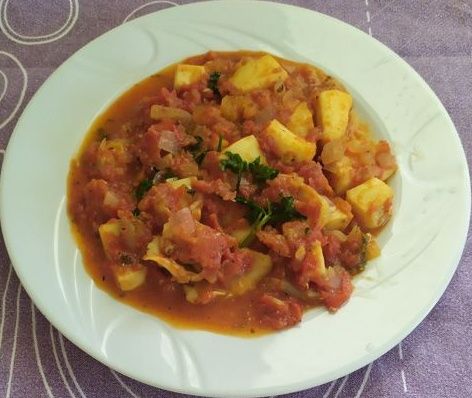 | 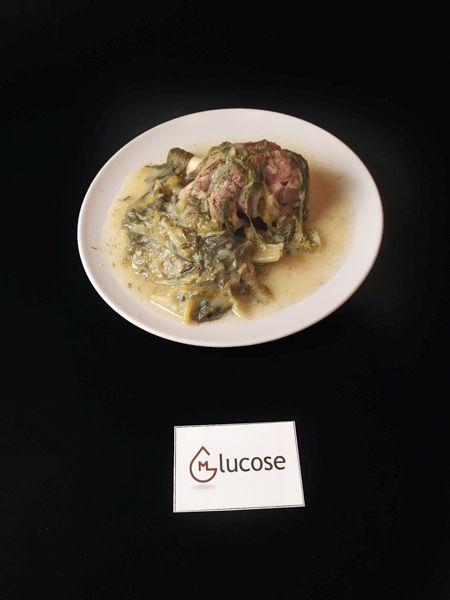 | 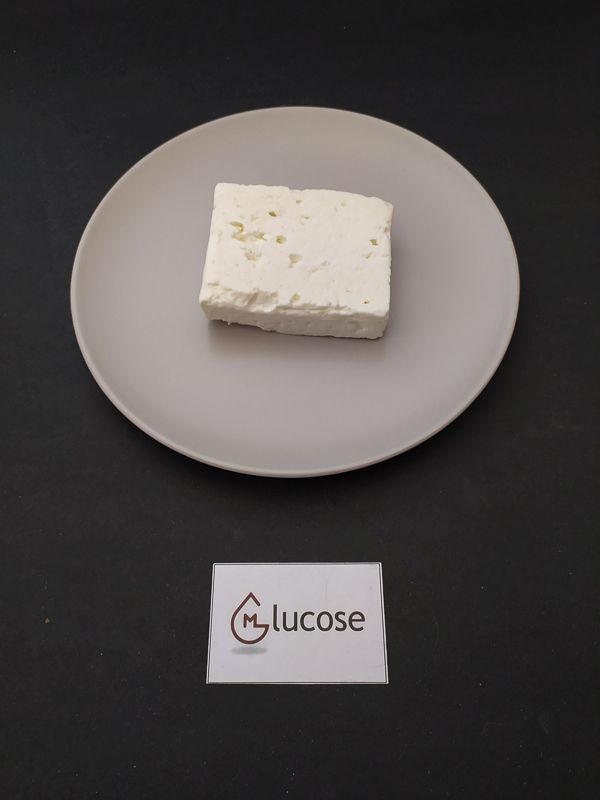 | 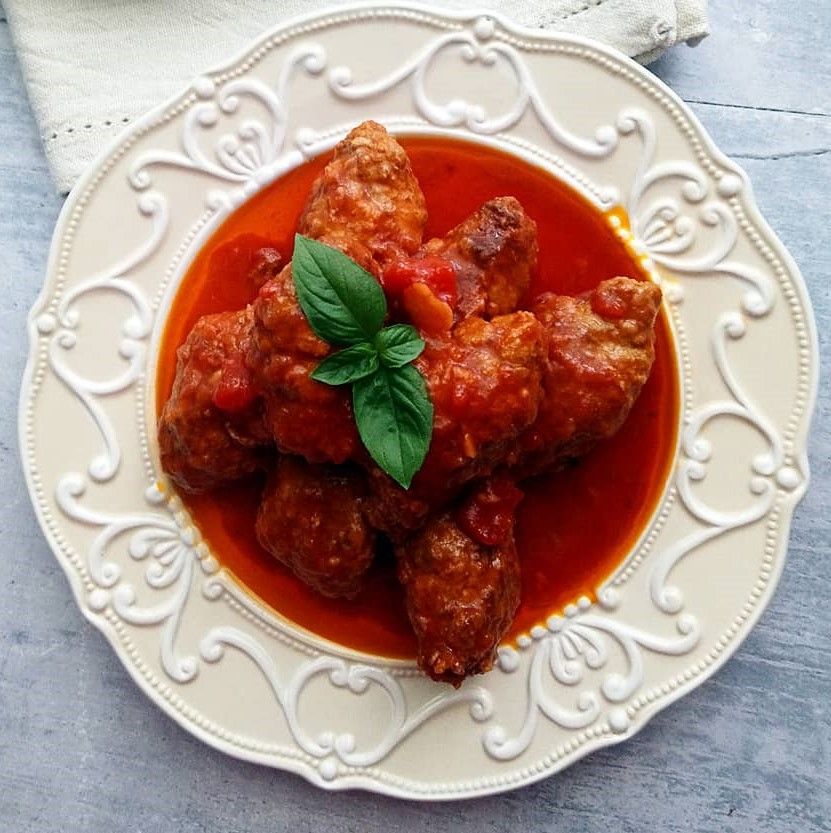 | 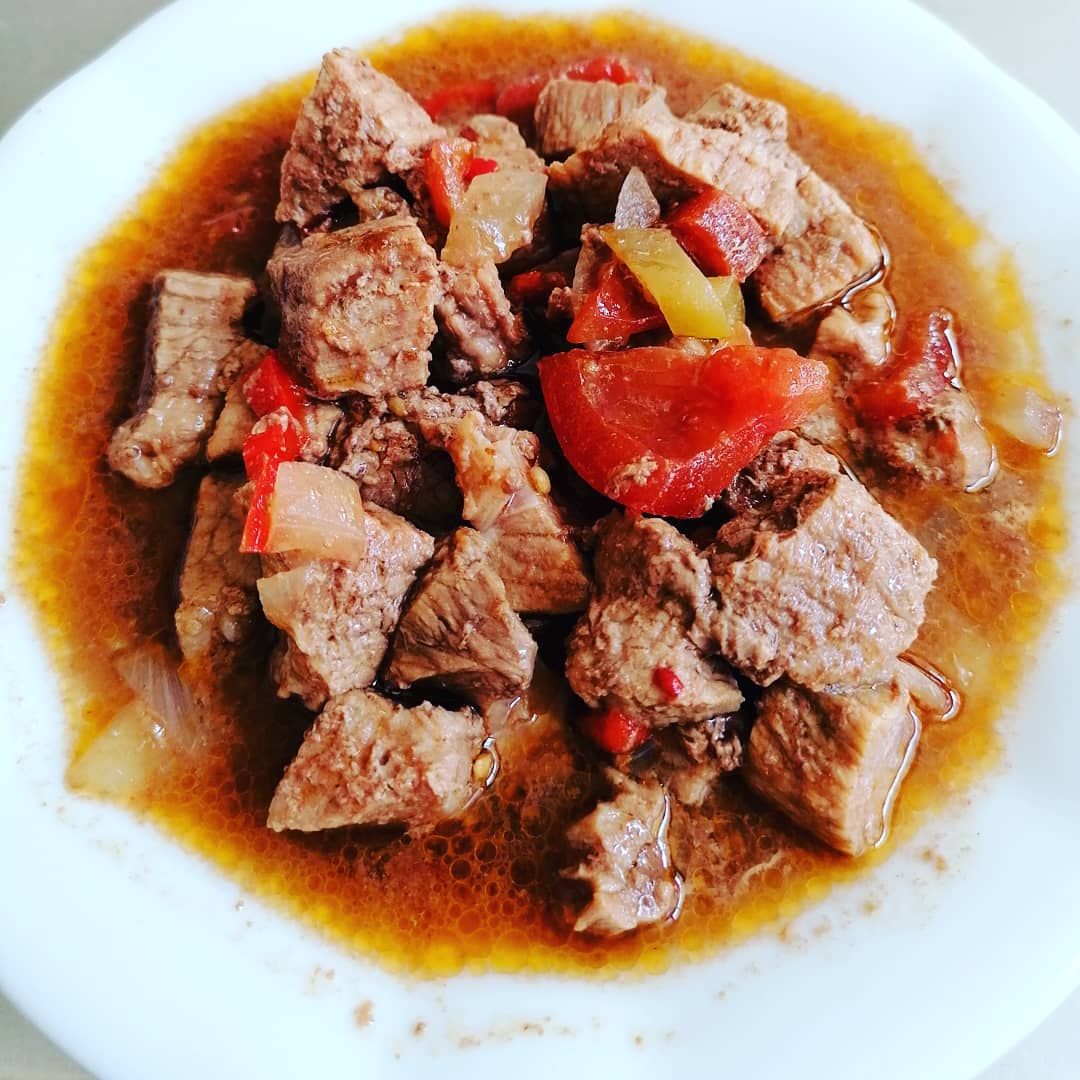 |

Fig. 1 Food images of the proposed MedGRFood dataset. From the left to right: cabbage rolls, almond cream cake, dolmades, baked sardines, fava, boiled beef with vegetables, imam bayildi, calamari with spinach, moussaka, pastitsio, pork souvlaki, rabbit stew with onions, pita gyro, ravani, shrimp saganaki, tzatziki, galaktoboureko, spinach and cheese pie, Greek salad, cuttlefish in red sauce, lamb fricassee, Greek feta cheese, soutzoukakia and tas kebab.


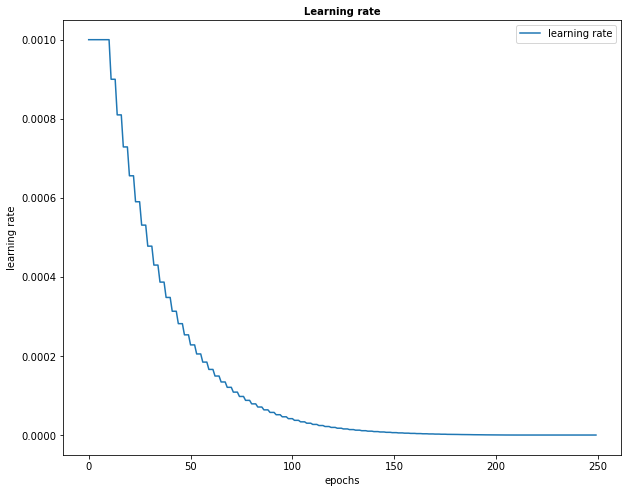


Fig. 2 Learning rate schedule.


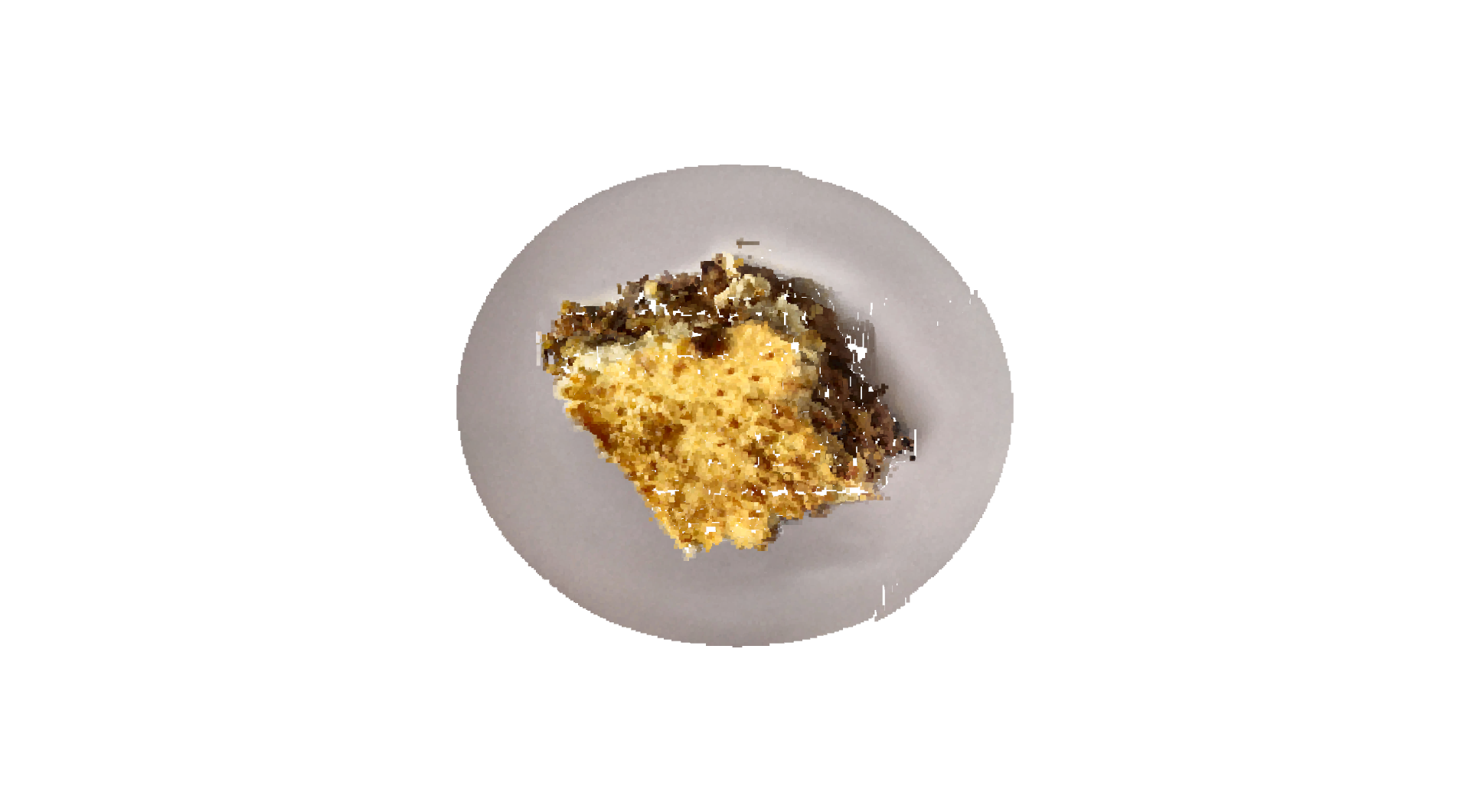


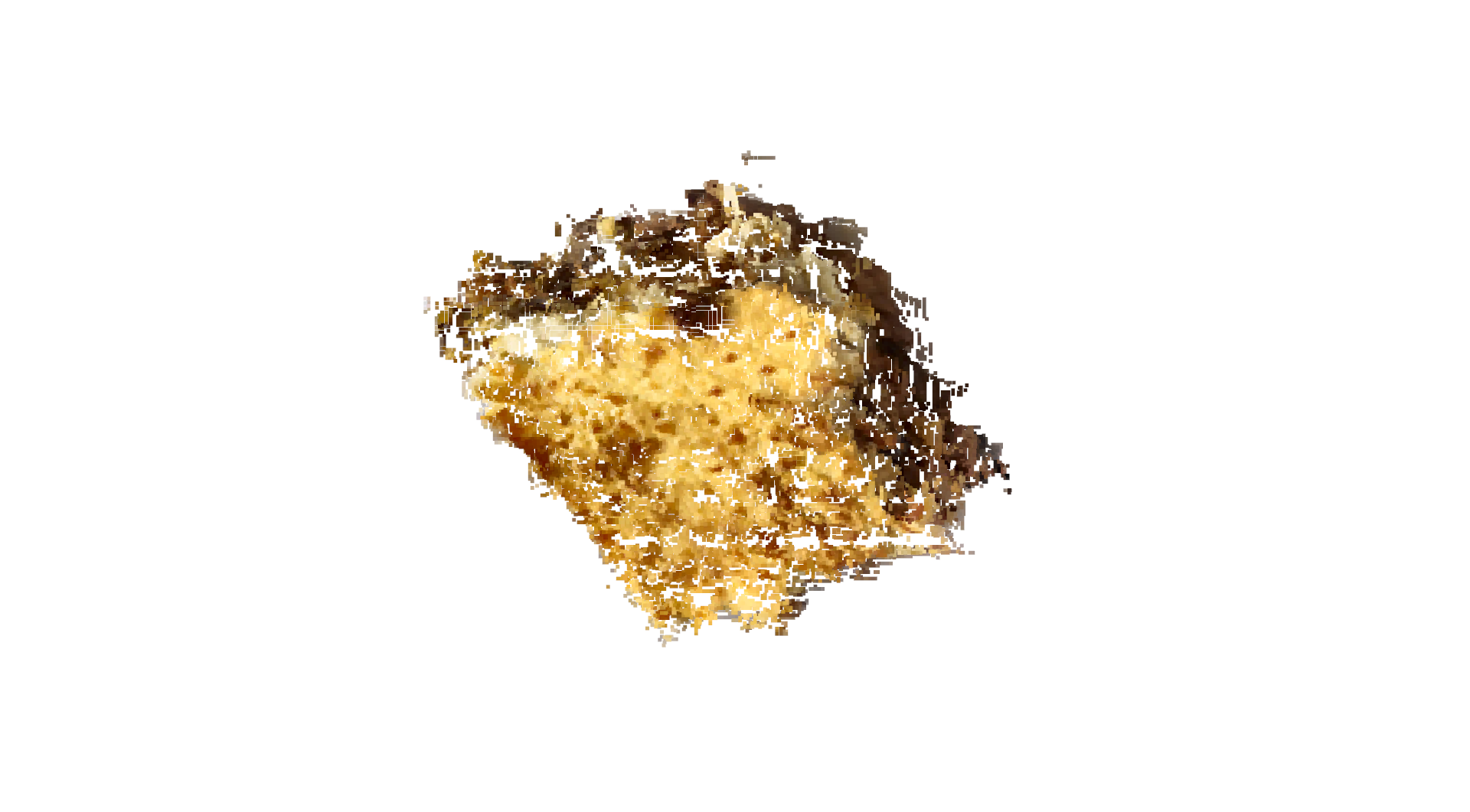


Fig. 3 A high resolution version of point cloud images.

Table I Top-5 Dishes With The Higher MAPE

| Food dish | Food category | MAPE (%) |
| --- | --- | --- |
| Fish roe dip | Miscellaneous food products | 21.3 |
| Eggplant salad | Miscellaneous food products | 20.3 |
| Semolina halvah | Grain or grain products | 18.9 |
| Beet salad | Miscellaneous food products | 17.7 |
| Tzatziki | Miscellaneous food products | 17.4 |

Table II Top-5 Dishes With The Lower MAPE

| Food dish | Food category | MAPE (%) |
| --- | --- | --- |
| Beefsteak | Meat or meat products | 5.1 |
| Pork souvlaki | Meat or meat products | 5.1 |
| Pork chop | Meat or meat products | 5.2 |
| Pasta Bolognese | Grain or grain products | 5.5 |
| Pasta with cheese | Grain or grain products | 5.6 |
